# Proteomic maps of breast cancer subtypes

**DOI:** 10.1038/ncomms10259

**Published:** 2016-01-04

**Authors:** Stefka Tyanova, Reidar Albrechtsen, Pauliina Kronqvist, Juergen Cox, Matthias Mann, Tamar Geiger

**Affiliations:** 1 Department of Proteomics and Signal Transduction, Max Planck Institute of Biochemistry, Martinsried 82152, Germany; 2 Computational Systems Biochemistry, Max Planck Institute of Biochemistry, Martinsried 82152, Germany; 3 Department of Biomedical Sciences, University of Copenhagen, Copenhagen 2200, Denmark; 4 Department of Pathology, University of Turku, 20520 Turku, Finland; 5 Department of Human Molecular Genetics and Biochemistry, Sackler Faculty of Medicine, Tel Aviv University, Tel Aviv-Yafo 6997801, Israel

## Abstract

Systems-wide profiling of breast cancer has almost always entailed RNA and DNA analysis by microarray and sequencing techniques. Marked developments in proteomic technologies now enable very deep profiling of clinical samples, with high identification and quantification accuracy. We analysed 40 oestrogen receptor positive (luminal), Her2 positive and triple negative breast tumours and reached a quantitative depth of >10,000 proteins. These proteomic profiles identified functional differences between breast cancer subtypes, related to energy metabolism, cell growth, mRNA translation and cell–cell communication. Furthermore, we derived a signature of 19 proteins, which differ between the breast cancer subtypes, through support vector machine (SVM)-based classification and feature selection. Remarkably, only three proteins of the signature were associated with gene copy number variations and eleven were also reflected on the mRNA level. These breast cancer features revealed by our work provide novel insights that may ultimately translate to development of subtype-specific therapeutics.

## Abstract (web summary)

Breast cancers have been extensively studied at the genomic and transcriptomic levels in the hope of tailoring therapeutic regimens. Here the authors generate deep coverage proteomes from several clinical breast cancer samples, and use machine learning techniques to uncover biological processes altered in specific cancer subtypes.

## Graphical abstract

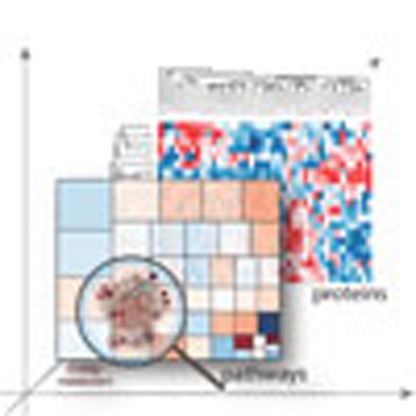

Breast cancer has been extensively studied at the genomic and transcriptomic levels to attain novel cancer classification that can alter therapeutic regimens^1^. The three main classical subtypes are defined by expression of the oestrogen receptor (ER), progesterone receptor (PR; ERPR positive breast cancer) and the epidermal growth factor receptor *ErbB2/Her2* (Her2 positive). The triple negative (TN) form (where none of the three markers is expressed) has an especially poor prognosis. More recently, unbiased approaches such as messenger RNA (mRNA) and gene copy number variation analyses identified novel classes based on the entire molecular profile. Initially, Perou *et al.* profiled gene expression patterns of dozens of breast tumours and identified the so called ‘intrinsic subtypes' of breast cancer, which have been reinforced in multiple studies with some modifications^2^,^3^,^4^. These subtypes matured into four accepted subtypes: Luminal A, Luminal B, Her2-enriched and basal-like breast cancer. While they do not perfectly reflect the clinical subtypes, most luminal tumours are ER/PR-positive, most Her2-enriched ones harbour the gene amplification, and most basal tumours are triple negative. Recently a large scale, integrated genomic-transcriptomic study further divided these subtypes into 10 clusters^5^, however, these have not yet been clinically accepted.

Taking a protein-based approach, we here use quantitative proteomics to examine the functional networks within the established breast cancer subtypes. We reasoned that analysis at the protein level, rather than genes and transcripts, may more directly reflect cellular functions. In a comparison to genomic data, we and others have previously shown a low correlation between the copy numbers of the gene in the genome and the relative change at the protein levels, meaning that many genomic variations are not or only partially translated to the protein level^6^,^7^. In addition, the correlation between mRNA and protein levels was also found to be far from perfect, thus examination of the mRNA alone does not necessarily reflect the active cellular functions^8^,^9^. Genome scale quantitative proteomic analysis is only now becoming possible due to multiple advances in the underlying MS technology, computational algorithms and biochemical technologies. For instance, high resolution, high speed mass spectrometers, in combination with advanced computational methods can now provide deep proteome coverage with high confidence in protein identification^10^. For accurate quantification we use the Stable Isotope Labelling with Amino Acids in Cell Culture technology (SILAC)^11^, which involves metabolic labelling of cells with lysine and arginine. The peptides generated by tryptic digestion are labelled in a ‘light' (normal isotopic) or ‘heavy' (stable isotope labelled) form and each of these peptide doublets contributes to protein quantification. We have expanded the use of SILAC to tumour tissues with the development of the super-SILAC technique, in which we use a combined lysate of different SILAC-labelled breast cancer cell lines as an internal standard for accurate quantification^12^. We further developed a protein extraction method for formalin-fixed paraffin-embedded (FFPE) tissue samples with little or no effect on trypsin digestion efficiency and peptide identification^13^. These developments now make it realistic to attempt system-wide quantitative proteomics of archived tumour samples. Here we applied the unbiased analysis of tumour proteomes to examine the potential of high-resolution mass spectrometry-based proteomics to clinical breast cancer research and to discover novel cancer regulators and subtype-specific biological processes.

## Results

### Proteomic profiling of breast cancer tumour samples

**Figure 1:**
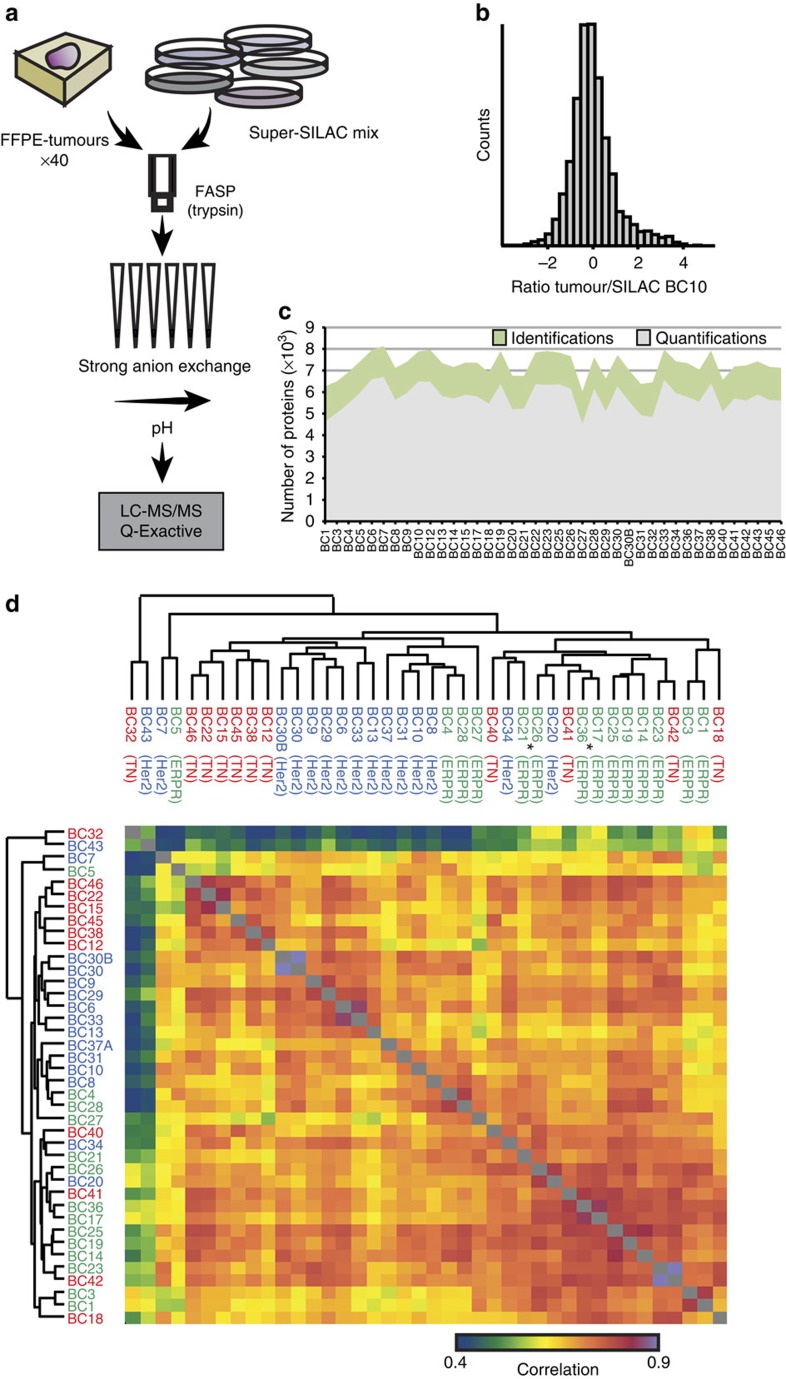
Super-SILAC-based quantitative proteomics of breast cancer clinical samples. (**a**) Proteomics workflow involved combination of the super-SILAC mix with FFPE tumour samples, followed by FASP digestion with trypsin, peptide fractionation and analysis on the Q Exactive MS. (**b**) Ratio distribution between the tumour proteome (of one representative tumour) and the super-SILAC mix showed overall narrow distribution that enables accurate ratio determination. (**c**) Plot shows the number of identified and quantified proteins in each tumour sample. (**d**) Hierarchical clustering of Pearson correlations of breast cancer samples shows high diversity between tumour samples, with only partial co-clustering of samples of the same classical subtype. Two triple-positive tumours are marked with *.

We analysed a panel of forty breast cancer samples consisting of 14 oestrogen receptor and/or progesterone receptor positive cases, 15 Her2 positives and 11 TNs. Initial subtype assignment was performed upon patient diagnosis using immunohistochemistry and fluorescence *in situ* hybridization FISH (Supplementary Table 1). Of the total set, 37 were ductal carcinomas and three lobular; most tumours were stage I–II, grades 2–3 and none were treated before tumour excision. Only two tumours expressed both *Her2* and *ER*, and therefore may be considered luminal B; but since this number is insufficient to analyse as a separate subtype, we classified it as ERPR in our downstream analyses. For the proteomic analysis we made use of two recently developed technologies: the first, FFPE-filter aided sample preparation (FASP), enables protein extraction from FFPE tissues^13^. The second, super-SILAC, uses a mixture of SILAC-labelled cells as a spike-in standard, thus enabling accurate quantification of the tumour proteomes^12^. We macrodissected tumour regions that are rich in cancer cells, extracted the proteins from the tumours, combined them with the breast cancer super-SILAC mix and then trypsin digested them using the FASP protocol^14^ (Fig. 1a). We wished to obtain a very deep proteomic coverage at acceptable measurement times and therefore generated six peptide fractions using strong anion exchange chromatography in a StageTip format^15^. Peptides were then measured on the quadrupole Orbitrap high-resolution mass spectrometer (Q Exactive)^16^ followed by data analysis in MaxQuant^17^. As previously shown for super-SILAC^12^, in all samples >90% of peptide ratios towards the internal standard were within a fivefold range (Fig. 1b). Technically, these ratios can be much more accurately determined than large ratios since in most cases both SILAC partners are well-above the noise level of the MS spectra. As a result, quantification relative to the super-SILAC mix provides high accuracy of quantification from the MS scans. Our analysis provided the largest breast tumour proteomic data set to date with a total of 157,544 identified sequence-unique peptides (Supplementary Data 1) and 10,135 identified proteins with 1% false discovery rate (FDR) both on the peptide-spectrum match and protein levels (Supplementary Data 2). On average, we identified >7,000 proteins in each sample (Fig. 1c), spanning 8 orders of magnitude of signal intensity (Supplementary Fig. 1). Interestingly, 95% of the proteins were within a much narrower abundance range of four orders of magnitude, and these still include important transcription factors such as *JUN* and *ATF2* (Supplementary Fig. 1). Our results constitute a systems wide, quantitative view of the proteomes of the clinical samples, which served as the basis for further downstream computational analysis and biological interpretation.

The high quantitative accuracy of the super-SILAC technology^12^ prompted us to investigate proteome differences within and between subtypes based on global protein expression levels. We first examined the correlation between samples to determine whether simple, unsupervised analysis would be sufficient to separate the classical breast cancer subtypes. Overall, Pearson correlations between the tumour proteomes were between 0.41 and 0.86 with an average correlation of 0.66. Unsupervised clustering of the correlations formed an interesting structure of tumour similarity at the proteomic level. Similarly to the genomics case^2^,^4^, they did not simply cluster into the three classical subtypes, but rather formed partial and mixed clusters (Fig. 1d). These results suggest that the variability among breast cancer subtypes detected by proteomics is influenced by other factors than the subtypes. These could for instance be owing to individual differences in the microenvironment, or heterogeneity among the cancer cells themselves, which interfere with cancer subtype signals.

### Proteomics data cover known mRNA gene sets

**Figure 2:**
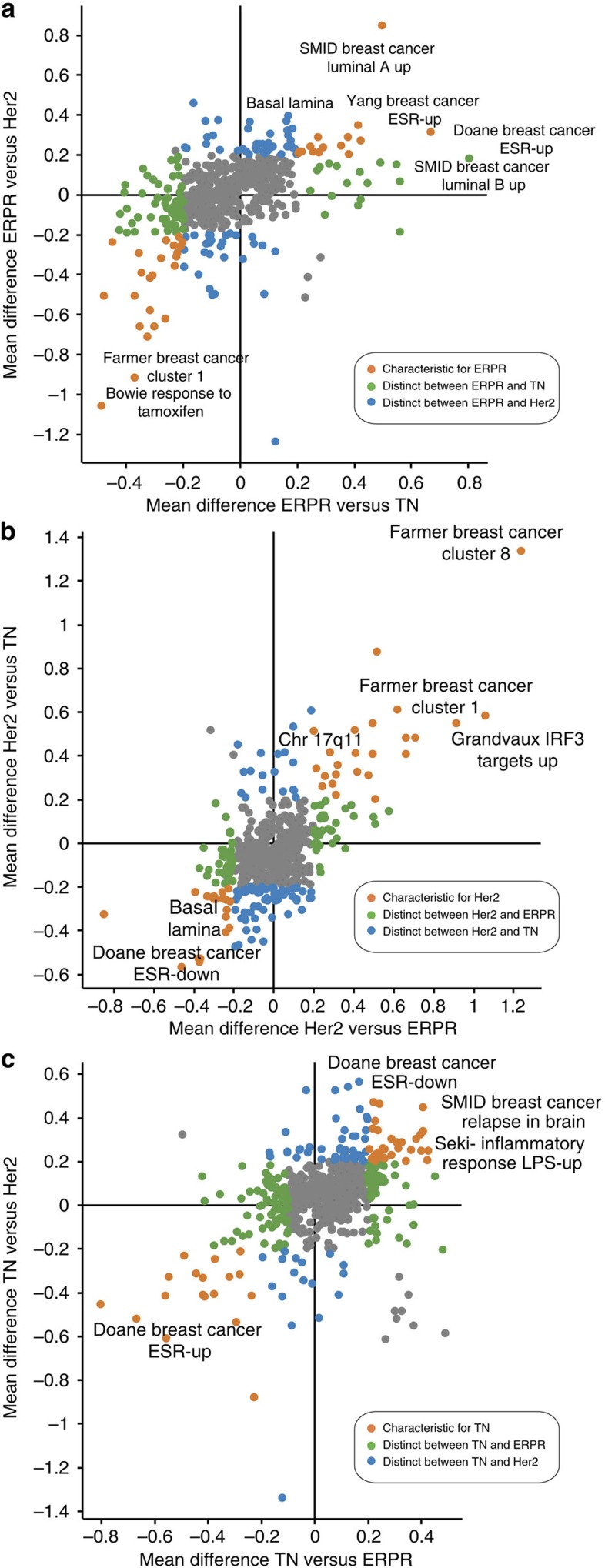
Molecular signatures database (MSigDB) analysis. Enrichment analysis of molecular signatures from the MSigDB database was performed on the average protein fold changes between the ER (**a**), Her2 (**b**) and TN (**c**) subtypes. Molecular signatures that differentiate one subtype with respect to both other subtypes are shown in orange, whereas categories that differ only between two subtypes are shown in blue or green. Complete list is given in Supplementary Data 3.

We examined whether the proteomics data captures previously described differences between breast cancer subtypes using the molecular signatures database (MSigDB) resource of gene sets^18^. For each protein, we calculated the mean difference of expression between the subtypes and performed annotation matrix analysis in Perseus, a statistical module in the MaxQuant software (http://www.perseus-framework.org/). Using the non-parametric Mann–Whitney test, the analysis allows for identification of proteins that belong to the same gene set (for example, associated with a disease condition) and whose expression is overall higher or lower in a particular breast cancer subtype as compared with the others. A total of 706 gene sets were significantly enriched (*P* value<0.5e-2) and we plotted them according to the quantitative difference of the proteins in each set between the subtypes (Fig. 2 and Supplementary Data 3). We found clear enrichment of multiple breast cancer-related gene sets derived from transcriptomics in the appropriate subtype. For example, ERPR tumours were enriched in the Doane *et al.*^19^, Yang *et al.*^20^ and Smid *et al.*^21^ gene sets that had been found to be high in ER-positive or luminal tumours. Furthermore, ERPR tumours showed low expression of genes that were downregulated by Tamoxifen according to Bowie *et al.*^22^. The Her2 graph showed the most marked enrichment of the gene set of Farmer-cluster 8 (ref. 23), which refers to the amplicon of *Her2* on chromosome 17—providing clear independent validation of our workflow. In addition, we found high levels of ‘*IRF3* targets'^24^ and Farmer-cluster 1 (ref. 23) both of which highlight involvement of interferon signalling and potentially immune response in this subtype. The gene sets with comparatively high protein expression in TN tumours included ‘breast cancer relapse in brain_up', which is typical of basal tumours, from Smid *et al.*^21^ and response to LPS from Seki *et al.*^25^. The LPS signature includes the *EGF* receptor and *CD44*, which are known TN markers and may be associated with increased immune infiltrates, which are often seen in TN tumours^26^. Thus, our proteomic analysis captures known molecular signatures of breast cancer subtypes, while revealing connections to additional mRNA signatures.

Next, we examined whether the proteomic data can segregate the tumour samples into the four intrinsic subtypes that were established using mRNA data. The PAM50 signature determines the tumour subtype based on the mRNA expression levels of 50 proteins^27^. Of the 50 genes in this signature, we identified 41 corresponding proteins, 21 of which had quantitative data in >70% of all samples. Hierarchical clustering of this signature showed co-expression of proteins representing the same intrinsic subtype (Supplementary Fig. 2). For example, *FOXA1*, *MLPH*, *NAT1*, *MAPT* and *BAG1* are expressed in luminal tumours according to published mRNA profiles and also co-clustered in our data, with higher expression levels in most ERPR tumours. Her2 positive tumours were clearly distinguished from the others based on the high protein expression of the known markers *Her2* and *Grb7*. Overall, these analyses show multiple similarities between mRNA and protein levels for the PAM50 gene set, and highlight the potential to distinguish between classical breast cancer subtypes at the protein level.

### Functional discrimination between breast cancer subtypes

**Figure 3:**
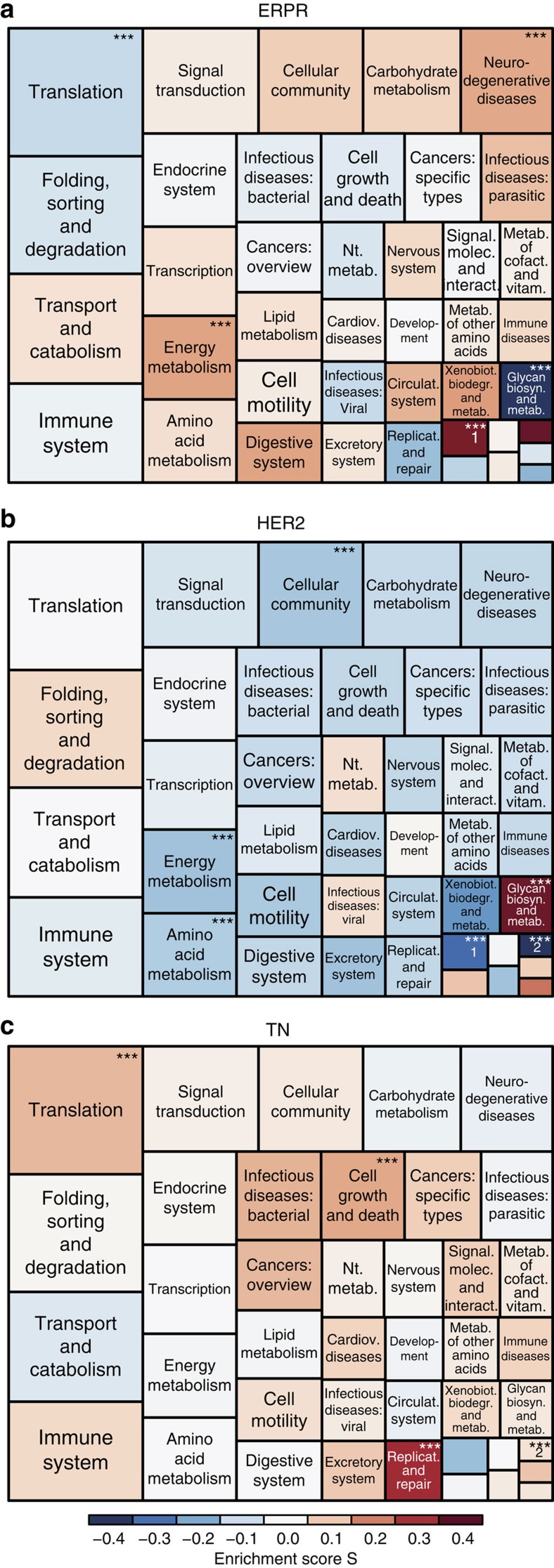
Tree-maps of subtype-specific KEGG categories. Enrichment of breast cancer subtype-specific KEGG pathways is shown for ERPR (**a**), Her2 (**b**) and TN (**c**) breast cancer subtypes. Each Tree-map includes all KEGG pathways mapped to a high hierarchical level. The size of the boxes corresponds to the number of proteins in that category and the color—to the enrichment score S, computed with the one dimensional enrichment test. Categories in red are characterized by higher average expression in the corresponding subtype, and in dark blue—by lower average expression. The categories that are significantly enriched at FDR 5% are indicated by ***. Category ‘1' stands for ‘global and overview maps' and category ‘2'—for ‘biosynthesis of other secondary metabolites'.

**Figure 4:**
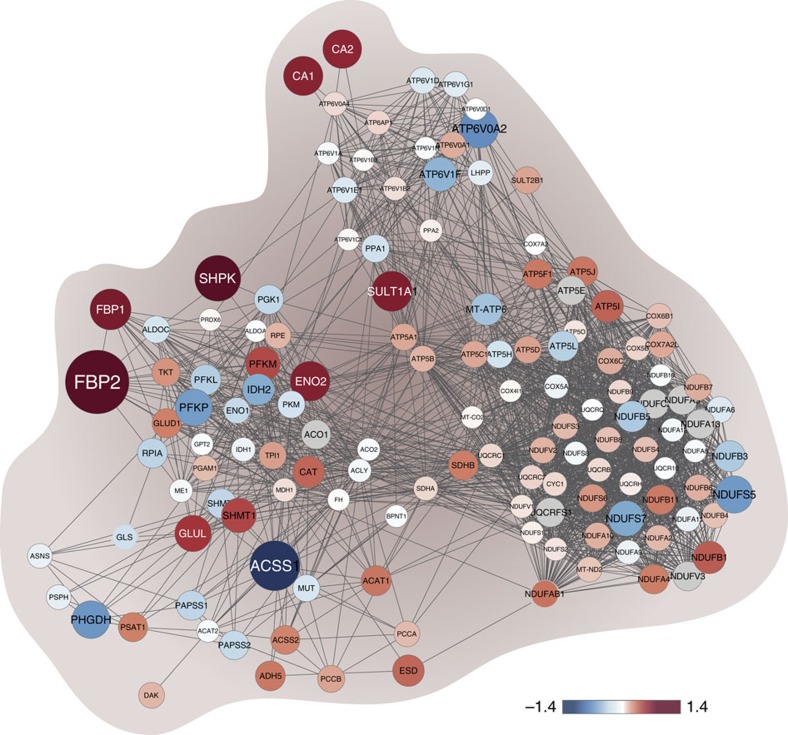
Energy metabolism protein–protein interaction network in ERPR tumours. Protein–protein interaction network of all proteins in our data set that mapped to the ‘energy metabolism' KEGG category was constructed in String (www.string-db.org). The color of the nodes correspond to the protein expression fold change between the ERPR and the two other subtypes; red indicates higher expression and blue—lower expression in the ERPR subtype. The size of the nodes corresponds to the absolute protein expression fold change.

We reasoned that analysis of the proteomic level could unravel coherent changes in cellular pathways, and identify key networks associated with each one of the subtypes. To that end, for each subtype we constructed functional tree-maps of KEGG pathways according to their inter-subtype differences (Fig. 3 and Supplementary Fig. 3). The tree-maps were divided into two hierarchical levels using the KEGG BRITE functional hierarchies. Each protein was assigned to the next two levels in the hierarchy; the immediate pathway level is represented in Supplementary Fig. 3 and the upper level in Fig. 3. Each tree-map includes all KEGG pathways in that hierarchical level, coloured according to their enrichment score and sized according to the number of proteins in that pathway. Highlighted are the pathways that showed statistically significant differences in their distribution and reflect the unique biology of each subtype. As an example, we found a significant increase in ‘energy metabolism' in ERPR and downregulation of this pathway in Her2 tumours (Fig. 3a and Fig. 3b, respectively). A protein–protein interaction network of members of this pathway shows higher expression levels of multiple proteins in ERPR tumours relative to the two other subtypes (Fig. 4). The network shows mild increase in multiple components of the electron-transport chain (*NDUF*, *UQCR*, *SDH* and *COX* subunits) and the ATP synthase complex (*ATP5* and *ATP6* subunits), associated with marked elevation in the cytosolic carbonic anhydrases 1 and 2 (*CA1* and *CA2*), potentially to buffer intracellular acidification induced by increased oxidative phosphorylation. Interestingly, multiple proteins linked to known cancer-associated metabolic pathways, such as glycolysis, serine synthesis (*PHGDH*), glutamine consumption (*GLS*), were lower in the ERPR tumours. In agreement, the most strongly elevated proteins in the entire network, *FBP2* and *FBP1*, are key enzymes in gluconeogenesis, which opposes glycolytic flux. Thus, the ‘energy metabolism' network reveals marked inter-subtype differences, and points to different metabolite utilization and synthesis to support the growth and survival of each of the subtypes.

TN tumours were characterized by elevated ‘replication and repair', ‘cell growth and death' and ‘translation' (Supplementary Fig. 4d,e,f). All of these pathways characterize rapidly growing tumours, one of the key features of TN tumours. The translation network shows higher levels of ribosomal proteins, ribosome biogenesis proteins, most markedly of *RCL1*, and the translation support machineries of tRNA-synthetases (*ARS* family) and nuclear pore complex components. The replication and growth networks pinpoint the marked increase in multiple cell cycle regulators and DNA replication proteins, such as MCM complex proteins, DNA polymerases, DNA damage response proteins, *CDK1*, *CDK2*, *CDK6* and *PCNA*. One of the key regulators, which is downregulated in this subtype, is the tumour suppressor *PTEN* thus supporting activity of the oncogenic *PI3K* pathway. *PTEN* is commonly deleted in TN tumours^28^, thus the proteomic data captures this feature and connects the network associated with it and reflects the known increased proliferative capacity of these tumours.

The Her2 subtype was characterized by reduced ‘amino acid and energy metabolism' (Supplementary Fig. 4c). This category includes proteins involved in amino acid, fatty acid, alcohol oxidation and more; and further reinforces the marked differences between the subtypes in the generation of cellular energy. For example, alcohol oxidation by alcohol dehydrogenase (*ADH*) and the subsequent reactions by aldehyde dehydrogenases (*ALDH*) were lower in Her2 tumours. Fatty acid oxidation, represented by acyl-CoA dehydrogenases (*ACAD*) was also reduced. While the pathway was generally lower in Her2 tumours, multiple proteins were actually upregulated. These upregulated enzymes are involved in diverse functions, such as proline metabolism (*PYCR1*,*2*, *PYCRL* and *PRODH*), methionine (*HNMT*) and tryptophan metabolism (*KMO*). The ‘cellular community' pathway consists of cytoskeletal proteins, extracellular matrix and cell adhesion molecules (Supplementary Fig. 4b). Similar to the metabolic pathways described above, as a whole, it was significantly downregulated in the Her2 subtype, but some of the network proteins were upregulated. Myosins (*MYH*) were overall reduced, as well as the extracellular protein family of laminins. In contrast, several collagens and fibronectin were upregulated in this subtype. This network further showed higher levels of thrombospondin (*THBS1* and *2*), which may mediate cell–matrix interaction. In the Her2 subtype, our most striking finding was the elevation in ‘glycan biosynthesis and metabolism', which includes proteins involved in glycosylation in the golgi, such as fucosyltransferase (*FUT8*), acetylgalactosaminyltransferase (*GALNT 2, 3, 6*), as well as proteins that are involved in glycan degradation in the lysosome, such as hexosaminidase (*HEXA*, *HEXB*), mannosidases (*MAN2B2*, *MAN1B1*, *MAN2A1*). All of these changes suggest marked differences in the glycosylation patterns in the Her2 subtype (Supplementary Fig. 4a). Such alterations have not yet been thoroughly investigated. Overall, these results highlight the main functional differences between breast cancer subtypes and reveal a molecular network associated with these functions.

Next, to address the statistical differences between subtypes we performed an analysis of variance (ANOVA) test (FDR 5%), which identified 62 significantly changing proteins between any of the three subtypes (Supplementary Fig. 5 and Supplementary Data 4). Hierarchical clustering of these proteins showed segregation into five main clusters, three of which include proteins that are specific to only one of the subtypes (more highly expressed), whereas the two others were shared between two subtypes. The TN clusters (TN and TN+Her2) include proliferative proteins (*MCM3* and *5*), translation related proteins (*RCL1*, *MRPS27*, *EIF2S2* and *EEF1G*) and metabolic enzymes (glutaminase and hexokinase 2), which reflect the higher dependence on glutamine and glucose of TN versus the other tumours. The Her2 clusters primarily show *Her2*, *Grb7* and *CDK12*, all of which are amplified on chromosome 17. In addition, it includes several chaperones (*DNAJA1*,*2*) and Golgi members (*FUT8*, *COG3*) that may be associated with the altered glycosylation patterns discussed above. Last, the ERPR clusters show significant increase in *Erk1* (*MAPK3*), which is unique to this subtype; *NDUFAB1*, a member of the electron-transport chain, and the transcriptional regulator *FOXA1*, which shows to be a major determinant of ER function^29^.

### Supervised identification of discriminative proteins

**Figure 5:**
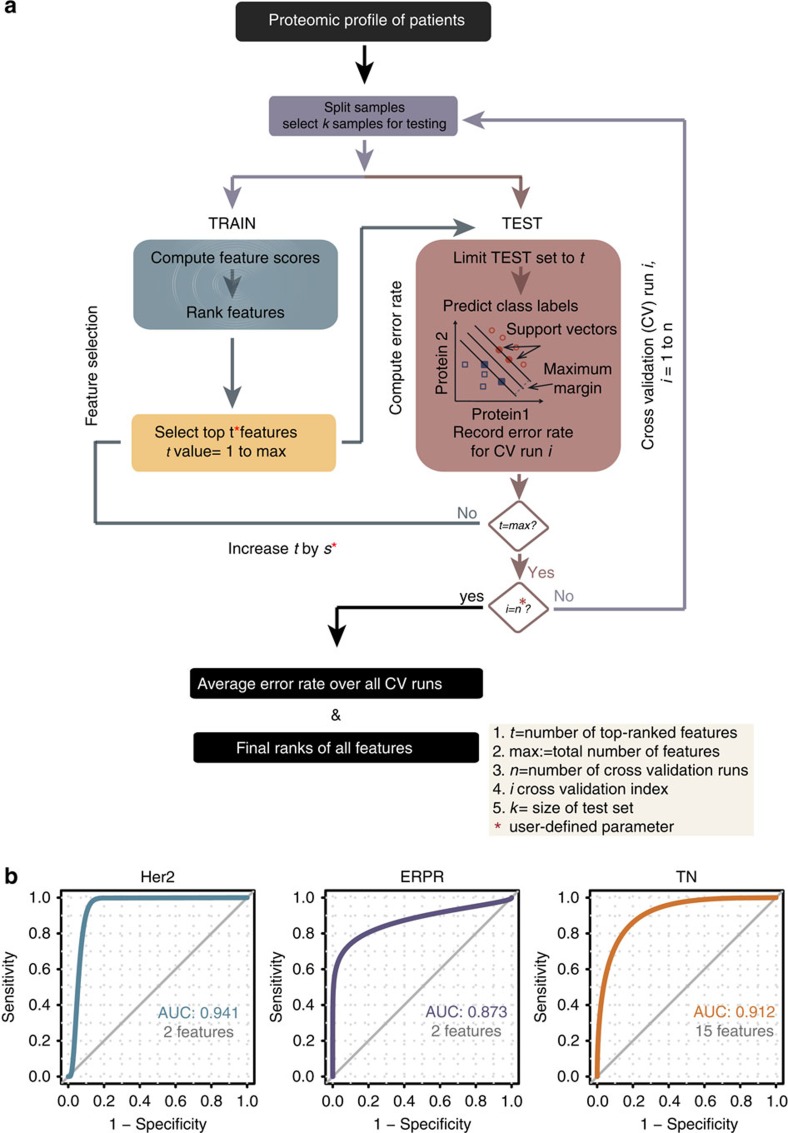
Computational workflow of feature selection in breast cancer patients. (**a**) To establish a predictive classifier of breast cancer subtypes, we developed a workflow that embeds classification and feature selection in a random sampling cross-validation procedure. In this framework, the user can define the fraction of samples to be randomly selected for the test set, as well as the number of such random samplings. The features in the training set are then ranked based on a pre-defined scoring scheme. Next, the predictive power of sets of ranked features of different sizes is computed in the test set and recorded for each cross-validation run. (**b**) Receiver operating characteristics (ROC) curves are given for each of the three predictors built using the optimal number of selected features. The performance of the predictors is shown by the area under curve (AUC) and ranges from 0.87 for the ERPR subtype to 0.94 for the Her2 positive subtype.

To generalize our proteomic findings and enable translation of the results towards clinical applications, we developed a computational framework for cancer subtype classification based on protein expression (Fig. 5a). It incorporates several machine learning methods for identification of subtype-specific proteins and consists of three main parts: classification, feature selection and cross-validation and is integrated into our Perseus software for data analysis (http://www.perseus-framework.org/). We employ Support Vector Machines (SVMs)^30^,^31^, a supervised learning technique in which we train a prediction model from the protein expression data using the information of the known subtypes. To apply the SVM algorithm to data sets that contain more than two classes we implemented a one-vs-rest approach. This resulted in three separate models, each one separating one breast cancer subtype from the other two.

Subtype-specific proteins were identified with a feature selection procedure that ranks the proteins according to their discriminative power. In the current work, we employed an ANOVA-based method, which uses univariate *P* values in the computation of feature ranks. This method performs well in the determination of proteins that are strong discriminators between the subtypes on their own, and results in small protein signatures.

To avoid overfitting and to ensure maximum generalizability of our results, we embedded feature selection in a rigorous cross-validation procedure where we estimated the predictive strength of the features on only a subset of the entire data. We ranked the features on a random subset and tested the prediction accuracy of differently sized sets of these features on the test set. Repeating this procedure many times on randomly selected training data increases the confidence in the relevance of the selected proteins.

### A signature of classical breast cancer subtypes

SVM classification with ANOVA-based feature selection embedded in a random sampling cross-validation procedure resulted in a small signature of 19 proteins (ERPR: 2 proteins, Her2: 2 proteins and TN: 15 proteins). Among these proteins, *Her2* and *Grb7* were positive markers of *Her2* positive tumours (highly expressed); for ERPR tumours, *MAPK3* (*Erk1*) and *EEF1G* were positive and negative markers, respectively; and the positive markers of TN tumours were *MCM5*, *STMN1*, *GLS*, *RCL1*, *C9ORF114* and *ENO1*. Receiver operating characteristic curves of the classification showed an area under the curve of 0.94 for Her2 tumours, 0.87 for ERPR and 0.91 for TN (Fig. 5b). As opposed to simply using the three receptors for classification (ER, PR and Her2), this classifier identifies positive markers of TN tumours, which further reflect the proliferative and metabolic characteristics of this subtype.

### Copy number variation and transcription of selected proteins

Acquired somatic copy number variations contribute to cancer initiation and progression. They can directly lead to abnormal protein expression as, for example, in the well-studied case of Her2, but the extent to which copy number variations affect corresponding protein levels is not generally known. Therefore, we investigated the degree to which alterations that are already encoded at the genome level affect the protein expression of the predictive signatures. In addition, we examined to what extent the proteomics differences are reflected at the transcript level.

**Figure 6:**
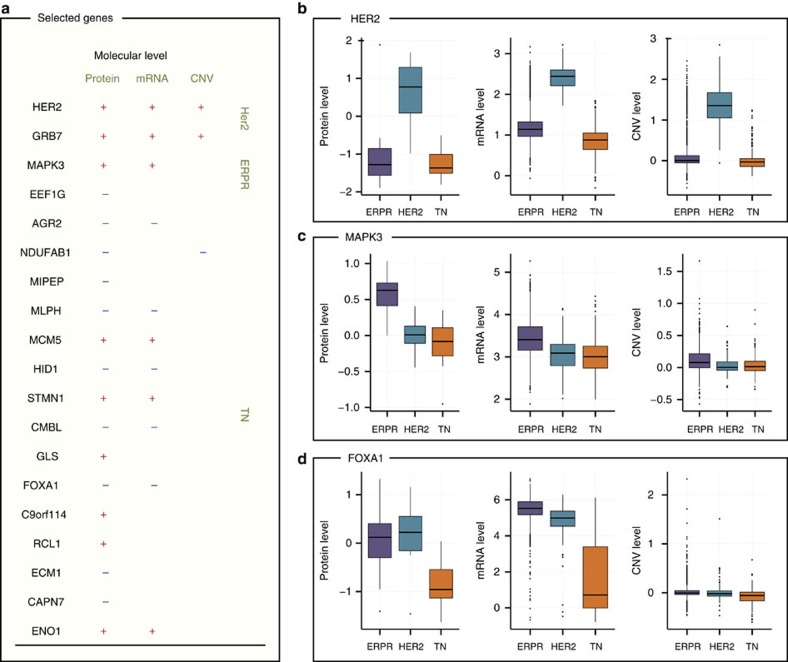
Comparison of SVM selected proteins on different expression levels. (**a**) Mapping of the 19 signature proteins to RNA and copy number variation (CNV) data from Curtis *et al.*^5^. Genes with statistically significant difference in expression at a given molecular level are marked by ‘+' and ‘−' if their expression at that level is higher or lower, respectively, in the particular subtype. Comparison of protein expression, mRNA expression and CNV distributions in each subtype are given for three selected proteins: Her2 (**b**), MAPK3 (**c**) and FOXA1 (**d**). All other proteins are presented in Supplementary Fig. 6. Quantitative information and local FDR values (*q* values) are given in Supplementary Data 4.

We mapped the 19 signature proteins to copy number variation and mRNA expression data from 1,992 breast cancer patients^5^ (Fig. 6 and Supplementary Fig. 6). We assigned these patient samples to one of the three major breast cancer subtypes based on the expression of the ER, PR and Her2. We then identified genome regions and transcripts that showed significant difference between the subtypes (see Methods). As expected, the two Her2 markers, namely, *Her2* and *Grb7*, which are known to be co-amplified in Her2 tumours were significantly different on all three levels. Only one additional marker, *NDUFAB1* was significant altered at the genomic level. Nine markers were found to change on both the mRNA and protein level (but not the gene level), including the positive ERPR marker *MAPK3* and the positive TN markers *MCM5*, *STMN1* and *ENO1*. Five negative markers showed lower levels in TN tumours on both protein and mRNA levels, including *AGR2*, *MLPH*, *HID1*, *CMBL* and, *FOXA1*. Beyond those markers, seven of the 19 signature proteins were exclusively regulated on the protein level.

Altogether, the proteomic results reveal network changes that are associated with clear functional differences between the subtypes. Furthermore, we show that the data can serve as the basis for the development of predictive subtype signatures.

## Discussion

This work is the deepest systems-wide quantitative proteomic study of breast cancer tumours to date and shows how proteomics can add to our understanding of subtype-specific key players and driving mechanisms. Accurately quantifying the patient proteomes was made possible by the development and combination of several technologies: super-SILAC-based quantification^12^, protein extraction from FFPE tissues^13^ and improvements in liquid chromatography–mass spectrometry analysis, which together allowed high proteome coverage with relatively short acquisition time. Just as importantly, the development of novel computational tools for the analysis of large proteomic data sets enabled the extraction of biologically meaningful differences between the tumour subtypes. Clearly, the recent advances in proteomics technologies now provide a solid platform for application of large-scale proteomic research to cancer subtypes. In the current study, we compared tissue samples from the three main breast cancer subtypes, ERPR, Her2 and TN. As examples of positive controls, the proteomic analysis unambiguously detected the high amplification and overexpression of the *Her2* and *Grb7* proteins, which are the known markers of Her2 tumours.

Previously, direct comparisons of mRNA and protein levels at a large scale have shown overall correlation of ∼0.6 in cancer cell lines^8^,^9^. While this implies a high level of agreement between these two levels of gene expression, it also provides evidence that expression levels of a large number of proteins are not directly predictable from mRNA levels. Despite these differences, our data showed enrichment of previously described mRNA-based signatures of these subtypes at the protein level. In particular, the proteomic data recapitulated the changes in mRNA levels of many but not all of the PAM50 genes. Remarkably, four well-described differentiating proteins in breast cancer subtypes, namely, *Her2*, *Grb7*, *FOXA1* and *MLPH*, were clearly selected in the PAM50 as well as in the proteomic signatures.

A comparison to publically available CNV data showed that only three proteins (*Her2*, *Grb7* and *NDUFAB1*) were regulated on the genomic level, and seven proteins from our signature were exclusively significant on the protein level. An important advantage of our signature is its ability to capture positive markers of TN tumours. Among those, we identified *MCM5*, *STMN1*, *RCL1* and *C9ORF114*, proteins that reflect the high proliferation rate of these tumours. Two additional positive markers, the glycolytic enzyme enolase (*ENO1*) and *GLS* reflect the unique metabolism of this subtype, which relies on glycolysis and glutaminolysis.

The construction of functional tree-maps revealed entire protein networks that are unique to each one of the subtypes. One of the most intriguing networks is related to energy metabolism, and highilighted the fundamental discrepancy between the subtypes. Primarily, the ERPR are predicted to have higher oxidative metabolism while the other subtypes show higher dependence on glucose and glutamine. One of the key proteins, shown here to be lower in ERPR, is phosphoglycerate dehydrogenase (*PHGDH*). A previous screen showed its importance to breast cancer cell growth^32^. Interestingly, that screen was performed in MCF10DCIS cells, which is a TN cell line. In agreement, *PHGDH* mRNA was found to be a marker of basal tumours as a part of the PAM50 signature. Thus our results further support the involvement of *PHGDH* in ER negative tumours. In addition, we found markedly higher levels of frucose bisphosphatases (*FBP1* and *2*), which catalyse the rate limiting step of gluconeogenesis in ERPR. Loss of *FBP1* has recently been associated with a ‘stem-cell' phenotype^33^, which is associated with TN or basal tumours^34^. Snail-induced reduction in *FBP1* expression leads to increased glycolysis and reduced oxidative phosphorylation^33^. Moreover, our results agree with previous mRNA studies that showed high *FBP1* expression in ERPR tumours^35^ and importantly, add the entire metabolic networks that are associated with reduced glycolysis and increased oxidative phosphorylation in ERPR tumours. These marked metabolic alterations are further supported by our previous metabolic modelling study of breast cancer, which showed that high *PHGDH* levels are associated with increased glutamine uptake, typical of ER negative tumours, while ER-positive ones present increased glutamine production and secretion^36^ (presented here by higher *GLUL* levels). Our results present for the first time the molecular evidence for the distinct metbolic paths that are used in ERPR tumours and in the TN and Her2 subtypes.

Our results also highlighted major signalling and replication differences between subtypes. In agreement with our findings at the proteome level, TN tumours commonly present *PTEN*-loss^28^, which leads to constitutive activity of the *PI3K* pathway. Most other components of the pathway, such as *AKT*, *PI3K* showed only minor proteomic differences between subtypes. In the future it would be interesting to investigate to what degree such alterations could be captured in the analysis of phosphorylation patterns of breast cancer subtypes. The reduced level of *PTEN* was associated with higher levels of multiple components of the cell cycle machinery, which support the overall higher proliferative capacity of the cells. Recently loss of *PTEN* in thyroid cancer been associated with metabolic remodelling of the cells through increased glucose addiction^37^. Further functional investigation may be able to find the exact mechanisms that link all of these TN features, namely, *PI3K* signalling, high ribosomes/translation, high dependence on glucose and glutamine and high proliferative capacity in a cancer subtype-specific network.

Interestingly, many of the identified proteomic inter-subtype differences showed relatively small fold-differences (below twofold). Presumably, low fold changes result from the heterogenous nature of breast cancers, which may include cells with varying receptor expression levels. As a result, analysis of macrodissected tissue may average cells with distinct phenotypes. These results highlight the necessity to use a very accurate quantification approach such as SILAC. Moreover, in the future, discrimination between the distinct sub-populations within single tumours may further shed new light on tumour classification and response to treatment.

In summary, we have here shown for the first time that global profiling of breast cancer clinical samples with high quantification accuracy is now possible and that it allows the attribution of biological processes to the different breast cancer subtypes. Our data provide molecular details of the key discriminating pathways between the subtypes, as well as a predictive signature that may be translatable towards clinical use, with the distinct advantage of having positive markers of TN tumours. In the future, analysis of post-translational modifications could highlight additional levels of regulation of these subtypes. We view this work as a first step in the integration of our proteomics technology into translational cancer research, which may help to develop novel breast cancer markers and identify potential therapeutic targets.

## Methods

### Sample assembly

FFPE tumour blocks were obtained from the Turku University Hospital, Turku, Finland. The use of these samples for research was approved by the ethical committee of the institute. Forty breast tumour samples were selected for analysis, including 14 ERPR tumours, 15 Her2 and 11 TN (Supplementary Table 1). To enrich for cancer cells in the samples and eliminate high concentrations of stromal proteins, highly cellular regions were selected based on hematoxylin and eosin staining and after pathological examination, these regions were punched out from the paraffin block.

### Sample preparation

Tumour samples were deparaffinized with two 5 min incubations in xylene, followed by two 5 min incubations with absolute ethanol. After removal of ethanol, samples were vacuum-dried and resuspended in lysis buffer containing 100 mM Tris HCl pH 7.5, 4% SDS and 100 mM DTT. Samples were briefly sonicated, and incubated for 1 h at 95 °C.

The super-SILAC mix was composed of HCC1599, MCF7, HCC1937 cells (purchased from the German Collection of Microorganisms and Cell Cultures, DSMZ), HCC2218 (purchased from the American Type Culture Collection, ATCC) and HMEC (purchased from Lonza). Super-SILAC mix was prepared less than a year after the purchase of cells. Cells were metabolically labelled with ^13^C_6_^15^N_4_-arginine (Arg-10) and l-^13^C_6_^15^N_2_-lysine (Lys-8). Labelled amino acids were purchased from Cambridge Isotope Laboratories. HCC1599, HCC2218 and HCC1937 cells were SILAC-labelled by culturing them in RPMI in which the natural lysine and arginine were replaced by Lys-8 and Arg-10 and supplemented with 10% dialyzed serum and antibiotics. MCF7 cells were grown in DMEM containing Lys-8 and Arg-10 instead of the natural amino acids and supplemented with 10% dialyzed serum and antibiotics. Human mammary epithelial cells (HMEC) were cultured in Gibco Defined Keratinocyte-serum free medium with Lys-8 and Arg-10 instead of the natural amino acids. Cells were cultured for ∼8 doublings in the SILAC medium to reach complete labelling. Sub-confluent cultures of HMEC, HCC1937 and MCF7 were lysed. HCC2218 and HCC1599, which grow in suspension, were lysed in a state of exponential growth. All cells were washed with PBS before lysis and lysed with a buffer containing 4% SDS, 100 mM Tris HCl (pH 7.6) and 100 mM DTT. Lysates were incubated at 95 °C for 5 min, and then briefly sonicated. Protein concentrations of cell and tissue lysates were determined by tryptophan fluorescence emission at 350 nm using an excitation wavelength of 295 nm. The measurements were performed in 8 M urea using tryptophan as the standard. Super-SILAC was prepared by combining equal protein amounts of each of the protein lysates.

### Protein and peptide processing

Equal protein amounts of the super-SILAC mix and each of the tissue samples were combined and trypsin digested using the FASP protocol^12^. Briefly, lysates were diluted 1:8 in 8 M urea in 0.1 M Tris HCl pH 8.0 and loaded onto 30 kDa microcon devices (Millipore). FASP procedure included the following steps: SDS buffer replacement with 8M urea buffer, protein alkylation with iodoacetamide and replacement of urea buffer with 50 mM ammonium bicarbonate. Sequencing grade trypsin was then added to the samples at a ratio of 1:50 (μg trypsin: μg protein) and incubated overnight at 37 °C. After digestion peptides were collected with two washes with 50 mM ammonium bicarbonate. Peptide concentrations were determined by ultraviolet-light absorption at 280 nm.

We fractionated the peptides of each of the samples into six fractions by strong anion exchange chromatography in a StageTip format as described previously^15^. Briefly, microcolumns were assembled by stacking six layers of Empore Anion Exchange disk (Varian) in 200-μl pipette tips. Column equilibration and elution was performed in Britton & Robinson buffer (20 mM phosphoric acid, 20 mM boric acid and 20 mM acetic acid). The buffer was titrated with sodium hydroxide to the following pH: 3, 4, 5, 6, 8 and 11 for subsequent elution. Eluted peptides were purified and concentrated on C18 StageTips. Sample preparation of all samples was performed simultaneously.

### MS analysis

Peptides were eluted from StageTips with 80% acetonitrile and 0.5% acetic acid (buffer B) and vacuum concentrated to reach a volume of 6 μl. Samples were separated on in-house made 30-cm-reverse phase columns (75 μm inner diameter, 1.8 μm ReproSil-Pur C18 beads) on an EASY-nLC nano high performance liquid chromatography system (Thermo Scientific). HPLC was coupled online via a nano-electrospray ion source to a Q Exactive mass spectrometer (Thermo Scientific). Peptides were loaded onto the column with buffer A (0.5% acetic acid) and separated with a 200-min water-acetonitrile gradient. Higher energy collisional dissociation fragmentation was performed for the top-10 peaks in each MS scan. Resolution of the MS scans was 70,000 (at *m*/*z*=200 Th) and 17,500 for the MS/MS scans. MS measurements for the different samples were randomized to eliminate technical artifacts.

### Computational analysis

Raw MS files were analysed with the MaxQuant software version 1.4.1.4 (ref. 17). MS/MS spectra were searched in the Andromeda search engine^38^ against the forward and reverse Human Uniprot database including the variable modifications methionine oxidation and N-terminal acetylation, and the fixed modification of carbamidomethyl cysteine. Parent peptide masses and fragment masses were searched with maximal initial mass deviation of 6–20 p.p.m., respectively. Mass recalibration was performed with a preceding Andromeda search with a mass window of 20 p.p.m. A first level of FDR filtration was done on the peptide-spectrum match level, and this was followed by a second level of FDR control on the protein level. Both filtrations were performed at a FDR of 0.01. These filtrations were done using a standard target-decoy database approach. When two proteins (isoforms and homologues with two Uniprot identifiers) could not be distinguished based on the identified peptides, these were merged by MaxQuant to one protein group.

### Bioinformatic analysis

The bioinformatics analysis was mainly performed using our in-house freely available software Perseus (www.perseus-framework.org). Analysis steps that were performed in the statistical analysis environment R (ref. 39) are described where applicable.

*MSigDB analysis*. The corresponding molecular signatures from the MSigDB^18^ were mapped to each protein in our data set. We employed a generalization of the one dimensional enrichment test^40^, named here as annotation matrix analysis. In this analysis, the distribution of mean differences of all proteins in a category is tested for a significant shift with respect to the global distribution of values in a particular subtype. The analysis results in the identification of categories (that is, molecular signatures) that show differential expression in a particular breast cancer subtype.

*Tree-maps*. KEGG pathway annotations for each protein group in our data set were inferred from UniProt matching by UniProt id. Using the KEGG BRITE functional hierarchies, each protein was further assigned to the next two levels in the hierarchy corresponding to that pathway. The mean difference between one of the subtypes and the other two was computed for each protein. Enrichment of a given category in each breast cancer subtype was computed with one dimensional annotation enrichment test as described above. The analysis employs the non-parametric Wilcoxon–Mann–Whitney test that uses rank sums and an enrichment score indicates if a category is enriched for high expression (the score is close to 1) or low expression (close to −1). Multiple hypothesis testing is performed using the Benjamini–Hochberg correction^41^ at 5% significance level. The R package ‘treemap'^42^ was used for plotting.

### Classification analysis

Feature selection: a computational platform based on SVMs^30^,^31^ was developed and employed for the identification of proteins that strongly discriminate between three different breast cancer subtypes. The core of the classification module relies on the libsvm implementation^43^ and adds on a complete analysis framework. Before classification, missing values were imputed in each sample by drawing random numbers from a normal distribution characterized by a specific downshift with respect to the available values distribution and a fixed s.d. This approach assumes that the missing values result from low abundant proteins. To assure maximum generalizability of the results the feature selection was embedded in a random sampling cross-validation procedure. The total number of patient samples was divided into train and test sets where the test set comprised 15% of the total data. The random sampling procedure was repeated 250 times. For each cross-validation run the features in the training set were ranked using ANOVA-based ranking method. In this method, *t*-tests were applied to each feature comparing its expression in each subtype with the rest of the samples and the resulting *P* values were used to rank the features.

On ranking different sets of features of increasing size were used to predict the breast cancer subtype of the samples in the test set. The accuracy for each cross-validation run and set of ranked features was calculated using SVMs as a classifier and recorded. The one-vs-rest implementation of the SVMs resulted in three separate ranked lists—one for each subtype. The optimal number of features, that is, the minimum number of proteins that classify the samples with the smallest error, was defined from the final accuracy curve, computed from averaging the accuracies over all cross-validation runs. Receiver operating characteristic curves were plotted and the area under the curve was computed with the pROC package^44^ in R.

### Copy number variations and differentially expressed genes

Copy number variation and gene expression data were obtained from Curtis *et al.*^5^ for 1,992 breast cancer patients. To identify differentially expressed genes and genome regions with differential copy number variation standard ANOVA tests implemented in Perseus were employed using permutation-based FDR^45^ and significance level of 5%.

## Additional information

**Accession codes:** The mass spectrometry proteomics data have been deposited to the ProteomeXchange Consortium46 via the PRIDE partner repository with the data set identifier PXD002619.

**How to cite this article:** Tyanova, S. *et al.* Proteomic maps of breast cancer subtypes. *Nat. Commun.* 7:10259 doi: 10.1038/ncomms10259 (2016).

## Supplementary Material

Supplementary Figures and Table Supplementary Figures 1-6 and Supplementary Table 1

Supplementary Data Set 1 Peptides file. A list of identified peptides and their quantification in the form of normalized ratios between the standard and the tissue sample. The subtype annotation is indicated for each sample. Additionally, the file contains information about the peptide sequence, length, mass, score and posterior error probability (PEP), with which the peptides were identified, and the protein groups to which they are assigned. Sample names labeled with Intensity L and H refer to the light and heavy SILAC counterparts, respectively. The intensity value is the sum of these intensity values.

Supplementary Data Set 2 Protein groups file. A list of identified protein groups and their quantification in the form of normalized

Supplementary Data Set 3 Enriched MSigDB categories in each breast cancer subtype. A list of molecular signatures from the MSigDB with differential expression between the breast cancer subtypes and the corresponding average protein fold changes between all subtype pairs.

Supplementary Data Set 4 List of ANOVA significant proteins. Differentially expressed protein groups between the three breast.

## References

[b1] Reis-Filho J. S. & Pusztai L. Gene expression profiling in breast cancer: classification, prognostication, and prediction. Lancet 378, 1812–1823 (2011). 22098854 10.1016/S0140-6736(11)61539-0

[b2] Perou C. M. *et al.* Molecular portraits of human breast tumours. Nature 406, 747–752 (2000). 10963602 10.1038/35021093

[b3] Sorlie T. *et al.* Repeated observation of breast tumour subtypes in independent gene expression data sets. Proc. Natl Acad. Sci. USA 100, 8418–8423 (2003). 12829800 10.1073/pnas.0932692100 PMC166244

[b4] TCGA. Comprehensive molecular portraits of human breast tumours. Nature 490, 61–70 (2012). 23000897 10.1038/nature11412 PMC3465532

[b5] Curtis C. *et al.* The genomic and transcriptomic architecture of 2,000 breast tumours reveals novel subgroups. Nature 486, 346–352 (2012). 22522925 10.1038/nature10983 PMC3440846

[b6] Geiger T., Cox J. & Mann M. Proteomic changes resulting from gene copy number variations in cancer cells. PLoS Genet. 6, e1001090 (2010). 20824076 10.1371/journal.pgen.1001090 PMC2932691

[b7] Zhang B. *et al.* Proteogenomic characterization of human colon and rectal cancer. Nature 513, 382–387 (2014). 25043054 10.1038/nature13438 PMC4249766

[b8] Nagaraj N. *et al.* Deep proteome and transcriptome mapping of a human cancer cell line. Mol. Syst. Biol. 7, 548 (2011). 22068331 10.1038/msb.2011.81 PMC3261714

[b9] Schwanhausser B. *et al.* Global quantification of mammalian gene expression control. Nature 473, 337–342 (2011). 21593866 10.1038/nature10098

[b10] Mann M., Kulak N. A., Nagaraj N. & Cox J. The coming age of complete, accurate, and ubiquitous proteomes. Mol. Cell 49, 583–590 (2013). 23438854 10.1016/j.molcel.2013.01.029

[b11] Ong S. E. *et al.* Stable isotope labeling by amino acids in cell culture, SILAC, as a simple and accurate approach to expression proteomics. Mol. Cell. Proteomics 1, 376–386 (2002). 12118079 10.1074/mcp.m200025-mcp200

[b12] Geiger T., Cox J., Ostasiewicz P., Wisniewski J. R. & Mann M. Super-SILAC mix for quantitative proteomics of human tumour tissue. Nat. Methods 7, 383–385 (2010). 20364148 10.1038/nmeth.1446

[b13] Ostasiewicz P., Zielinska D. F., Mann M. & Wisniewski J. R. Proteome, phosphoproteome, and N-glycoproteome are quantitatively preserved in formalin-fixed paraffin-embedded tissue and analyzable by high-resolution mass spectrometry. J. Proteome Res. 9, 3688–3700 (2010). 20469934 10.1021/pr100234w

[b14] Wisniewski J. R., Zougman A., Nagaraj N. & Mann M. Universal sample preparation method for proteome analysis. Nat. Methods 6, 359–362 (2009). 19377485 10.1038/nmeth.1322

[b15] Wisniewski J. R., Zougman A. & Mann M. Combination of FASP and StageTip-based fractionation allows in-depth analysis of the hippocampal membrane proteome. J. Proteome Res. 8, 5674–5678 (2009). 19848406 10.1021/pr900748n

[b16] Michalski A. *et al.* Mass spectrometry-based proteomics using Q Exactive, a high-performance benchtop quadrupole Orbitrap mass spectrometer. Mol. Cell. Proteomics 10, M111.011015 (2011). 21642640 10.1074/mcp.M111.011015 PMC3284220

[b17] Cox J. & Mann M. MaxQuant enables high peptide identification rates, individualized p.p.b.-range mass accuracies and proteome-wide protein quantification. Nat. Biotechnol. 26, 1367–1372 (2008). 19029910 10.1038/nbt.1511

[b18] Subramanian A. *et al.* Gene set enrichment analysis: a knowledge-based approach for interpreting genome-wide expression profiles. Proc. Natl Acad. Sci. USA 102, 15545–15550 (2005). 16199517 10.1073/pnas.0506580102 PMC1239896

[b19] Doane A. S. *et al.* An estrogen receptor-negative breast cancer subset characterized by a hormonally regulated transcriptional program and response to androgen. Oncogene 25, 3994–4008 (2006). 16491124 10.1038/sj.onc.1209415

[b20] Yang F. *et al.* Laser microdissection and microarray analysis of breast tumours reveal ER-alpha related genes and pathways. Oncogene 25, 1413–1419 (2006). 16261164 10.1038/sj.onc.1209165

[b21] Smid M. *et al.* Subtypes of breast cancer show preferential site of relapse. Cancer Res. 68, 3108–3114 (2008). 18451135 10.1158/0008-5472.CAN-07-5644

[b22] Bowie M. L. *et al.* Interferon regulatory factor-1 regulates reconstituted extracellular matrix (rECM)-mediated apoptosis in human mammary epithelial cells. Oncogene 26, 2017–2026 (2007). 17016442 10.1038/sj.onc.1210013

[b23] Farmer P. *et al.* Identification of molecular apocrine breast tumours by microarray analysis. Oncogene 24, 4660–4671 (2005). 15897907 10.1038/sj.onc.1208561

[b24] Grandvaux N. *et al.* Transcriptional profiling of interferon regulatory factor 3 target genes: direct involvement in the regulation of interferon-stimulated genes. J. Virol. 76, 5532–5539 (2002). 11991981 10.1128/JVI.76.11.5532-5539.2002 PMC137057

[b25] Seki E. *et al.* TLR4 enhances TGF-beta signaling and hepatic fibrosis. Nat. Med. 13, 1324–1332 (2007). 17952090 10.1038/nm1663

[b26] Desmedt C. *et al.* Biological processes associated with breast cancer clinical outcome depend on the molecular subtypes. Clin. Cancer Res. 14, 5158–5165 (2008). 18698033 10.1158/1078-0432.CCR-07-4756

[b27] Parker J. S. *et al.* Supervised risk predictor of breast cancer based on intrinsic subtypes. J. Clin. Oncol. 27, 1160–1167 (2009). 19204204 10.1200/JCO.2008.18.1370 PMC2667820

[b28] Saal L. H. *et al.* Recurrent gross mutations of the PTEN tumour suppressor gene in breast cancers with deficient DSB repair. Nat. Genet. 40, 102–107 (2008). 18066063 10.1038/ng.2007.39 PMC3018354

[b29] Hurtado A., Holmes K. A., Ross-Innes C. S., Schmidt D. & Carroll J. S. FOXA1 is a key determinant of estrogen receptor function and endocrine response. Nat. Genet. 43, 27–33 (2011). 21151129 10.1038/ng.730 PMC3024537

[b30] Boser B. E., Guyon I. M. & Vapnik V. N. in *Proceedings of the 5th Annual Workshop on Computational Learning Theory COLT'92* (ACM Press, Pittsburgh, PA, USA, (1992).

[b31] Vapnik V. N. The nature of statistical learning theory NY Springer (1995).

[b32] Possemato R. *et al.* Functional genomics reveal that the serine synthesis pathway is essential in breast cancer. Nature 476, 346–350 (2011). 21760589 10.1038/nature10350 PMC3353325

[b33] Dong C. *et al.* Loss of FBP1 by Snail-mediated repression provides metabolic advantages in basal-like breast cancer. Cancer Cell 23, 316–331 (2013). 23453623 10.1016/j.ccr.2013.01.022 PMC3703516

[b34] Ben-Porath I. *et al.* An embryonic stem cell-like gene expression signature in poorly differentiated aggressive human tumours. Nat. Genet. 40, 499–507 (2008). 18443585 10.1038/ng.127 PMC2912221

[b35] van 't Veer L. J. *et al.* Gene expression profiling predicts clinical outcome of breast cancer. Nature 415, 530–536 (2002). 11823860 10.1038/415530a

[b36] Jerby L. *et al.* Metabolic associations of reduced proliferation and oxidative stress in advanced breast cancer. Cancer Res. 72, 5712–5720 (2012). 22986741 10.1158/0008-5472.CAN-12-2215

[b37] Morani F. *et al.* PTEN deficiency and mutant p53 confer glucose-addiction to thyroid cancer cells: impact of glucose depletion on cell proliferation, cell survival, autophagy and cell migration. Genes Cancer 5, 226–239 (2014). 25221641 10.18632/genesandcancer.21 PMC4162142

[b38] Cox J. *et al.* Andromeda: A Peptide Search Engine Integrated into the MaxQuant Environment. J. Proteome Res. 10, 1794–1805 (2011). 21254760 10.1021/pr101065j

[b39] R Core Team. R: A language and environment for statistical computing. R Foundation for Statistical Computing, Vienna, Austria (2012). Available at www.R-project.org/.

[b40] Cox J. & Mann M. 1D and 2D annotation enrichment: a statistical method integrating quantitative proteomics with complementary high-throughput data. BMC Bioinform. 13, (Suppl 16): S12 (2012). 10.1186/1471-2105-13-S16-S12 PMC3489530 23176165

[b41] Benjamini Y. & Hochberg Y. Controlling the false discovery rate: a practical and powerful approach to multiple testing. J. R. Stat. Soc. 57, 289–300 (1995).

[b42] Tennekes M. & Jonge E. Top-down data analysis with treemaps. in Proceedings of the International Conference on Information Visualization Theory and Applications IVAPP Algarve, Portugal (2011).

[b43] Chang C. C. & Lin C. J. LIBSVM: a library for support vector machines. ACM Trans. Intell. Syst. Technol. 2, 1–27 (2011).

[b44] Robin X. *et al.* pROC: an open-source package for R and S+ to analyze and compare ROC curves. BMC Bioinform. 12, 77 (2011). 10.1186/1471-2105-12-77 PMC3068975 21414208

[b45] Tusher V. G., Tibshirani R. & Chu G. Significance analysis of microarrays applied to the ionizing radiation response. Proc. Natl Acad. Sci. USA 98, 5116–5121 (2001). 11309499 10.1073/pnas.091062498 PMC33173

