# Supplementary material for: Proteomic maps of breast cancer subtypes
Source: Nat Commun. 2016 Jan 4;7:10259. doi: 10.1038/ncomms10259 (PMC4725767; doi:10.1038/ncomms10259)
Supplement: Supplementary Figures and Table — Supplementary Figures 1-6 and Supplementary Table 1 [file ncomms10259-s1.pdf]

Supplementary Figure 1

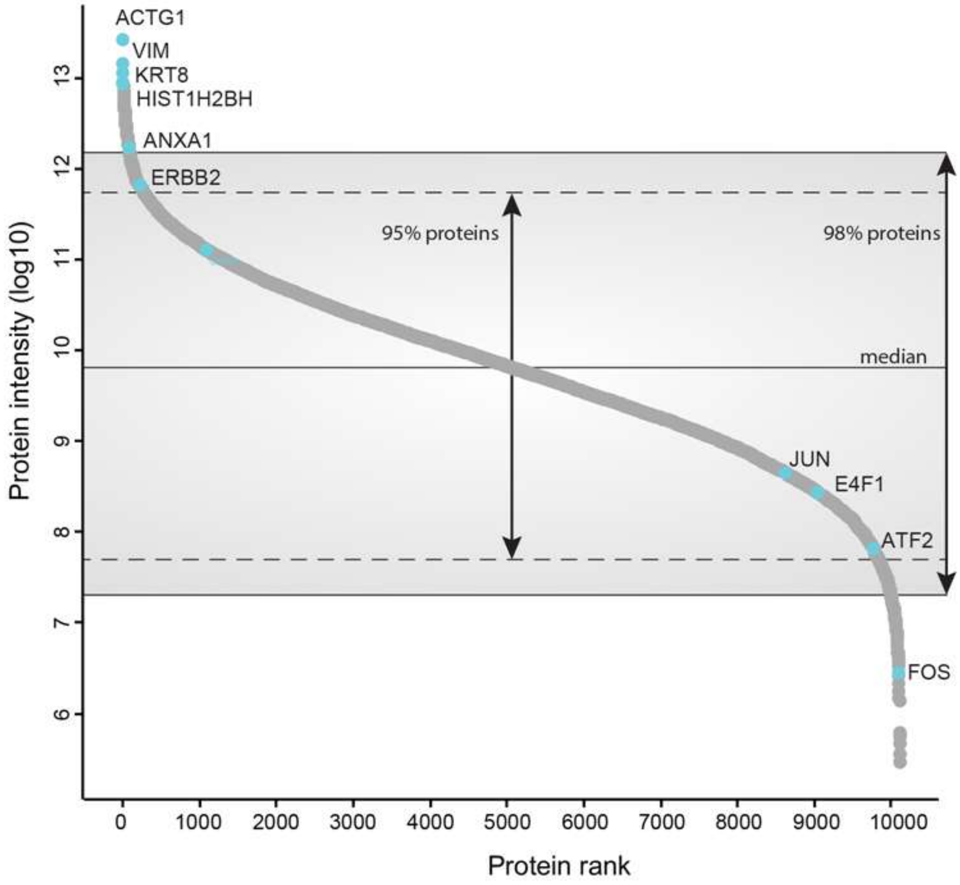

Supplementary Figure 1. Abundance curve of proteins. Ranking of protein abundances shows that the overall distribution of protein abundances spans eight orders of magnitude. Nevertheless, the vast majority of the proteins (95%) are within a range of four orders of magnitude.

Supplementary Figure 2

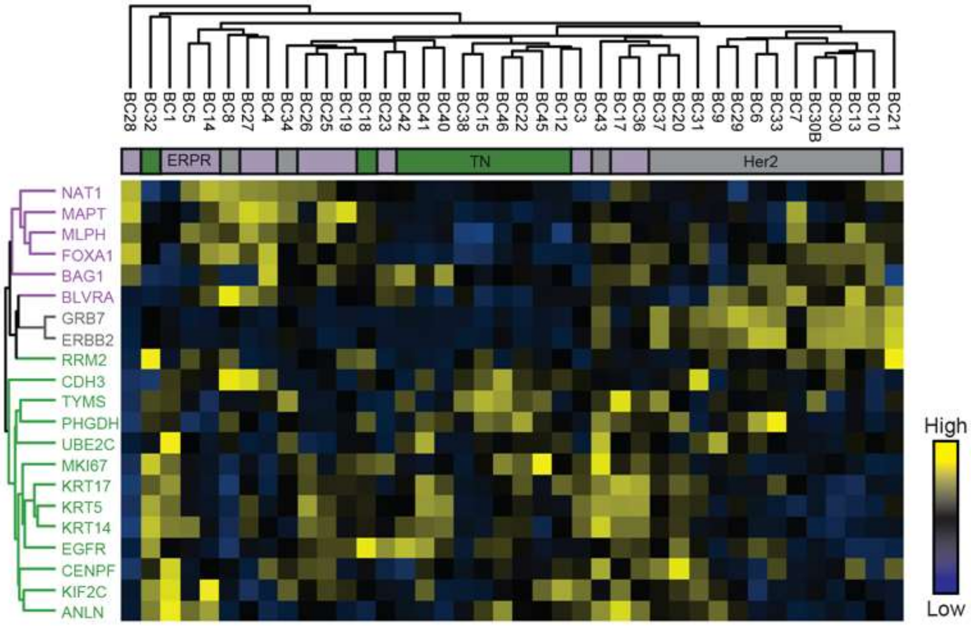

Supplementary Figure 2. Protein expression of the PAM50 genes. Hierarchical clustering of the protein levels (normalized ratio towards the super-SILAC mix). PAM50 genes enable partial segregation of classical subtypes on the protein level. Color code for gene names: purple- ER, gray- Her2, green-TN.

Supplementary Figure 3

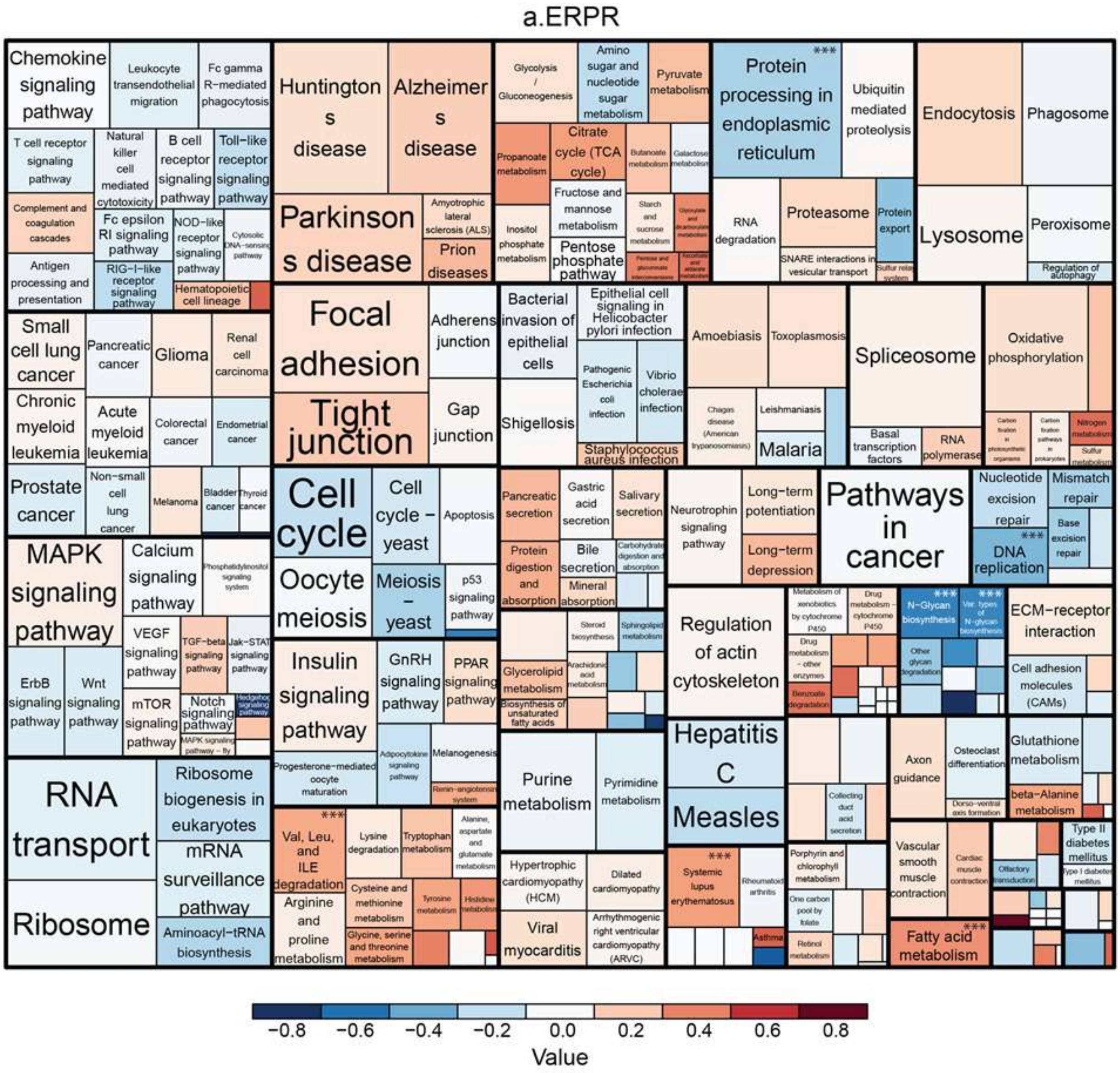

b.Her2

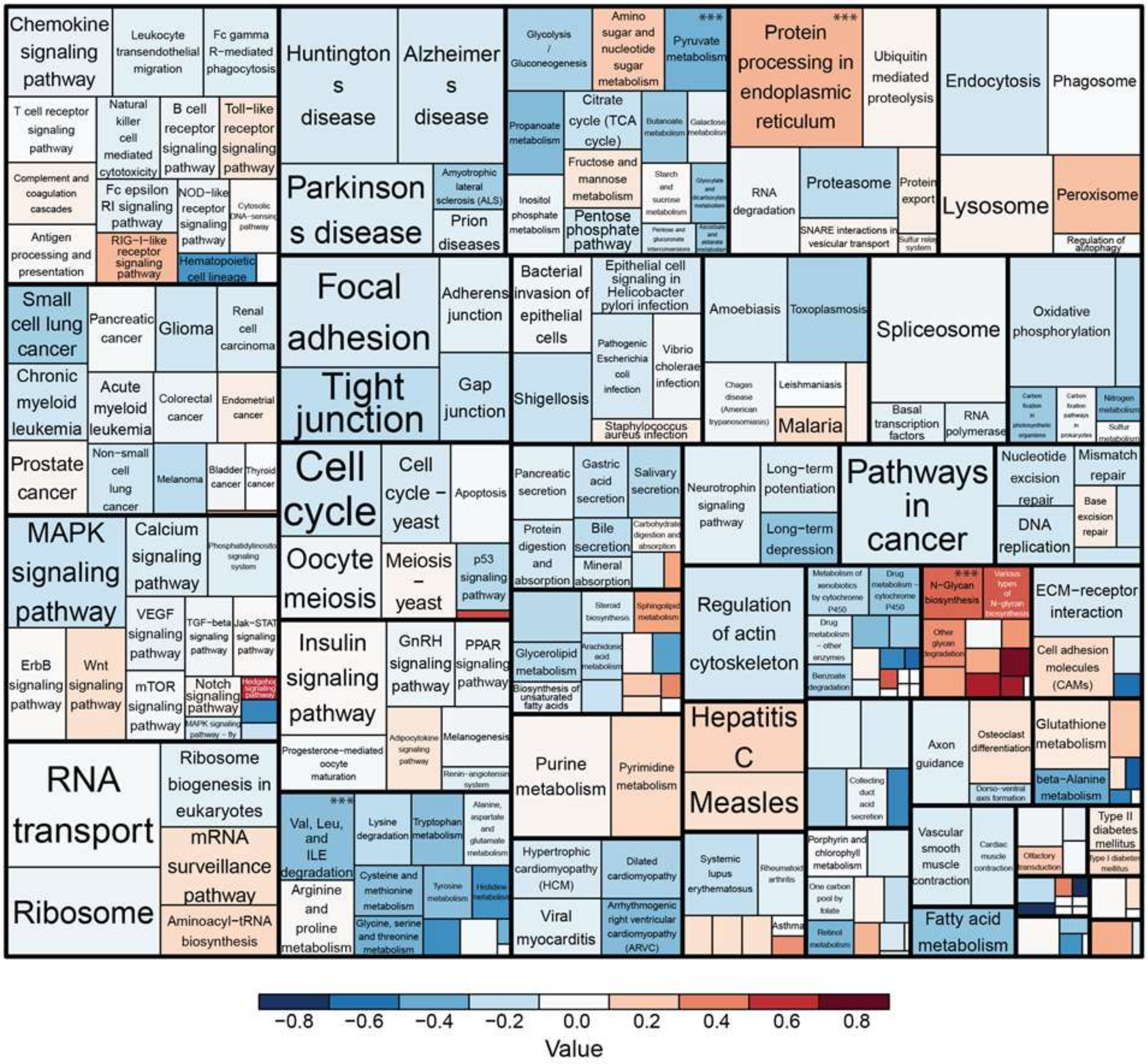
Chemokine signaling pathway
Leukocyte transendothelial migration
Fc gamma R-mediated phagocytosis
T cell receptor signaling pathway
Natural killer cell mediated cytotoxicity
B cell receptor signaling pathway
Toll-like receptor signaling pathway
Complement and coagulation cascades
Fc epsilon RI signaling pathway
NOD-like receptor signaling pathway
Cytosolic DNA-sensing pathway
Antigen processing and presentation
RIG-I-like receptor signaling pathway
Hematopoietic cell lineage
Huntington s disease
Alzheimer s disease
Parkinson s disease
Amyotrophic lateral sclerosis (ALS)
Prion diseases
Glycolysis / Gluconeogenesis
Amino sugar and nucleotide sugar metabolism
***
Pyruvate metabolism
Propanoate metabolism
Citrate cycle (TCA cycle)
Butanoate metabolism
Galactose metabolism
Inositol phosphate metabolism
Fructose and mannose metabolism
Starch and sucrose metabolism
Pentose phosphate pathway
***
Protein processing in endoplasmic reticulum
Ubiquitin mediated proteolysis
RNA degradation
Proteasome
Protein export
SNARE interactions in vesicular transport
Endocytosis
Phagosome
Lysosome
Peroxisome
Regulation of autophagy
Small cell lung cancer
Pancreatic cancer
Glioma
Renal cell carcinoma
Chronic myeloid leukemia
Acute myeloid leukemia
Colorectal cancer
Endometrial cancer
Prostate cancer
Non-small cell lung cancer
Melanoma
Bladder cancer
Thyroid cancer
Focal adhesion
Adherens junction
Tight junction
Gap junction
Bacterial invasion of epithelial cells
Epithelial cell signaling in Helicobacter pylori infection
Pathogenic Escherichia coli infection
Vibrio cholerae infection
Shigellosis
Staphylococcus aureus infection
Amoebiasis
Toxoplasmosis
Chagas disease (American trypanosomiasis)
Leishmaniasis
Malaria
Spliceosome
Basal transcription factors
RNA polymerase
Oxidative phosphorylation
Nitrogen metabolism
Sulfur metabolism
MAPK signaling pathway
Calcium signaling pathway
Phosphatidylinositol signaling system
VEGF signaling pathway
TGF-beta signaling pathway
Jak-STAT signaling pathway
ErbB signaling pathway
Wnt signaling pathway
mTOR signaling pathway
Notch signaling pathway
Hedgehog signaling pathway
Cell cycle
Cell cycle - yeast
Apoptosis
Oocyte meiosis
Meiosis - yeast
p53 signaling pathway
Insulin signaling pathway
GnRH signaling pathway
PPAR signaling pathway
Progesterone-mediated oocyte maturation
Adipocytokine signaling pathway
Melanogenesis
Pancreatic secretion
Gastric acid secretion
Salivary secretion
Protein digestion and absorption
Bile secretion
Carbohydrate digestion and absorption
Mineral absorption
Steroid biosynthesis
Sphingolipid metabolism
Glycerolipid metabolism
Arachidonic acid metabolism
Biosynthesis of unsaturated fatty acids
Neurotrophin signaling pathway
Long-term potentiation
Long-term depression
Pathways in cancer
Nucleotide excision repair
Mismatch repair
Base excision repair
DNA replication
Regulation of actin cytoskeleton
Drug metabolism - other enzymes
Benzoate degradation
***
N-Glycan biosynthesis
Various types of N-glycan biosynthesis
Other glycan degradation
ECM-receptor interaction
Cell adhesion molecules (CAMs)
RNA transport
Ribosome biogenesis in eukaryotes
mRNA surveillance pathway
Ribosome
Aminoacyl-tRNA biosynthesis
***
Val, Leu, and ILE degradation
Lysine degradation
Tryptophan metabolism
Arginine and proline metabolism
Cysteine and methionine metabolism
Glycine, serine and threonine metabolism
Purine metabolism
Pyrimidine metabolism
Hypertrophic cardiomyopathy (HCM)
Dilated cardiomyopathy
Arrhythmogenic right ventricular cardiomyopathy (ARVC)
Viral myocarditis
Hepatitis C
Measles
Systemic lupus erythematosus
Rheumatoid arthritis
Asthma
Collecting duct acid secretion
Porphyrin and chlorophyll metabolism
One carbon pool by folate
Retinol metabolism
Axon guidance
Osteoclast differentiation
Glutathione metabolism
beta-Alanine metabolism
Dorso-ventral axis formation
Vascular smooth muscle contraction
Cardiac muscle contraction
Olfactory transduction
Type II diabetes mellitus
Type I diabetes mellitus
Fatty acid metabolism
−0.8
−0.6
−0.4
−0.2
0.0
0.2
0.4
0.6
0.8
Value

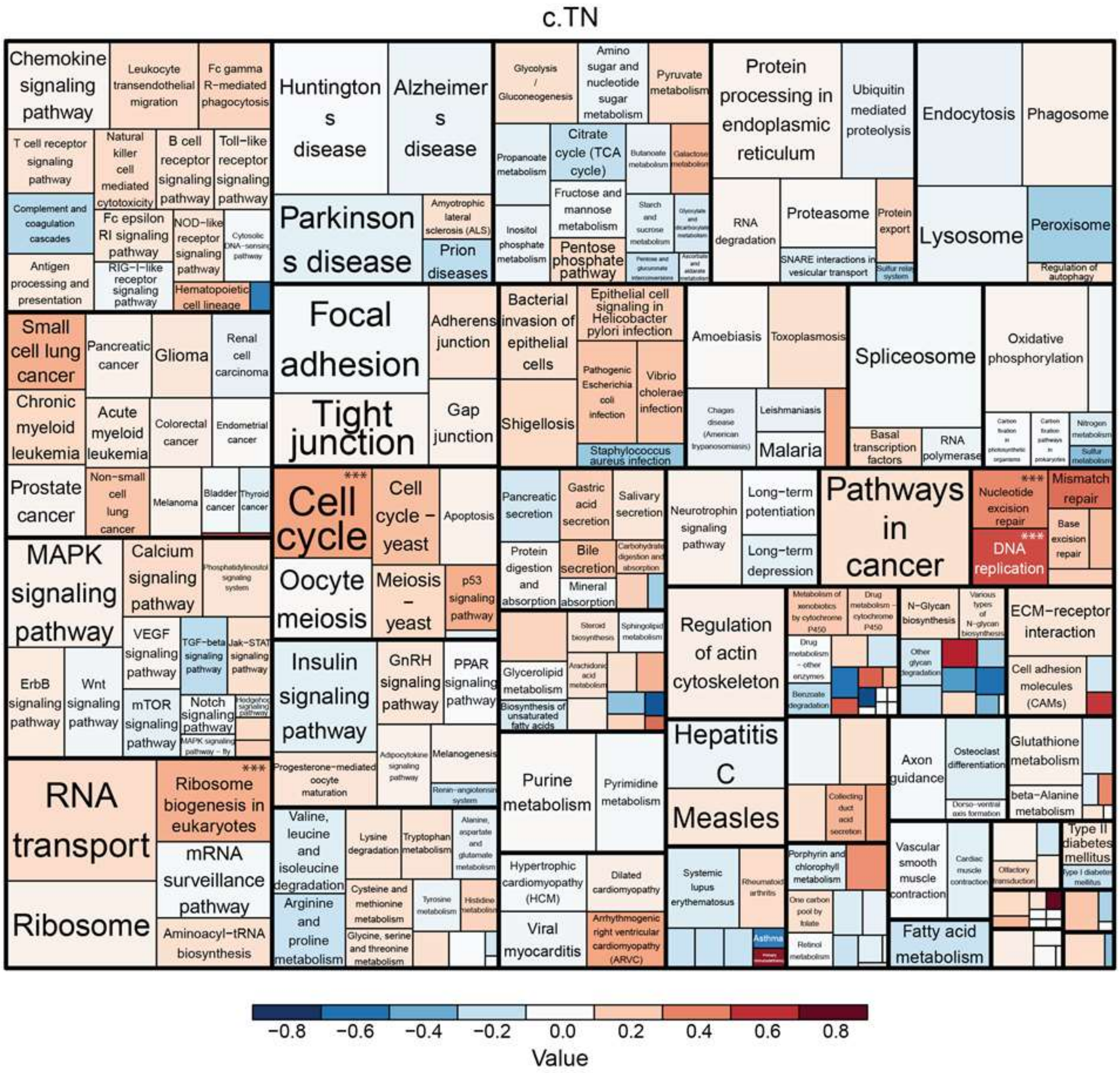

Supplementary Figure 3. Tree-maps of KEGG pathways. Enrichment of breast cancer subtype-specific KEGG pathways is shown for ERPR (**a**), Her2 (**b**), and TN (**c**) breast cancer subtypes. Each Tree-map includes all KEGG pathways. The size of the boxes corresponds to the number of proteins in that category and the color - to the enrichment score S, computed with the 1D enrichment test. Categories in red are characterized by higher average expression in the corresponding subtype, and in dark blue – by lower average expression. The categories that are significantly enriched at FDR 5% are indicated by ***.

Supplementary Figure 4

a. Glycan biosyntheis and metabolism

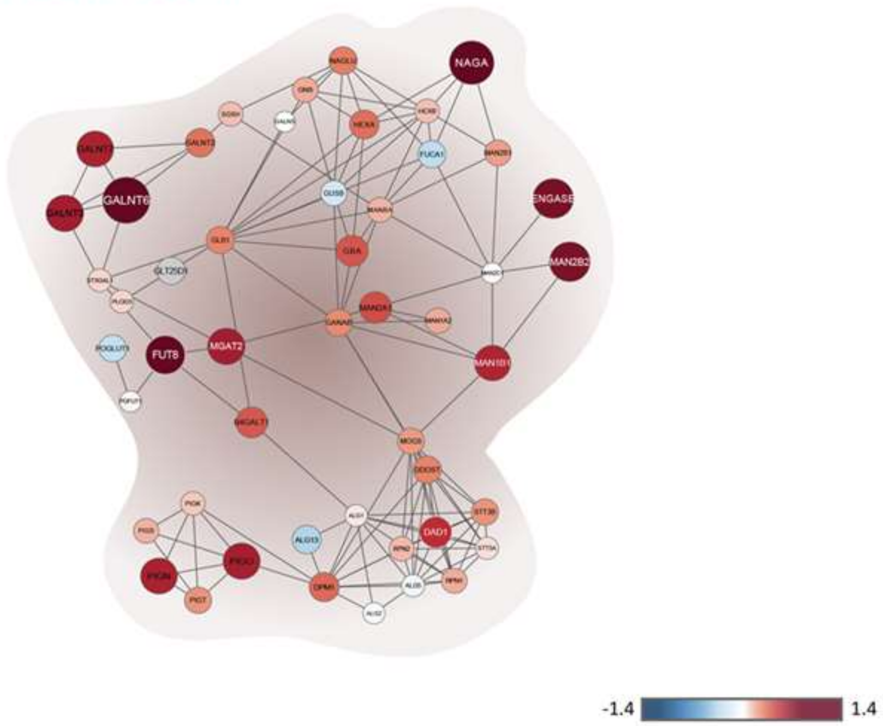

b. Cellular community

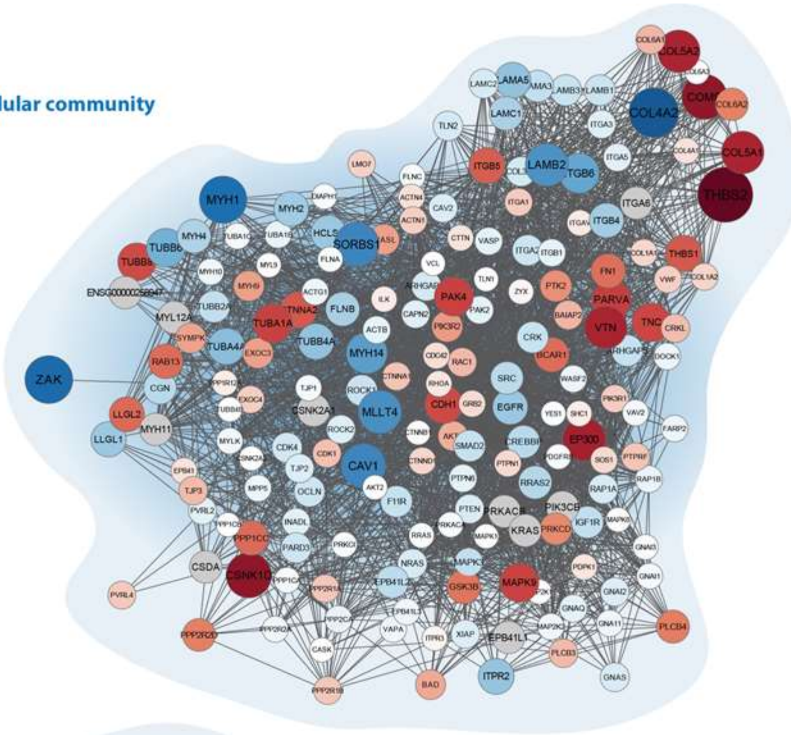

c. Amino acid metabolism

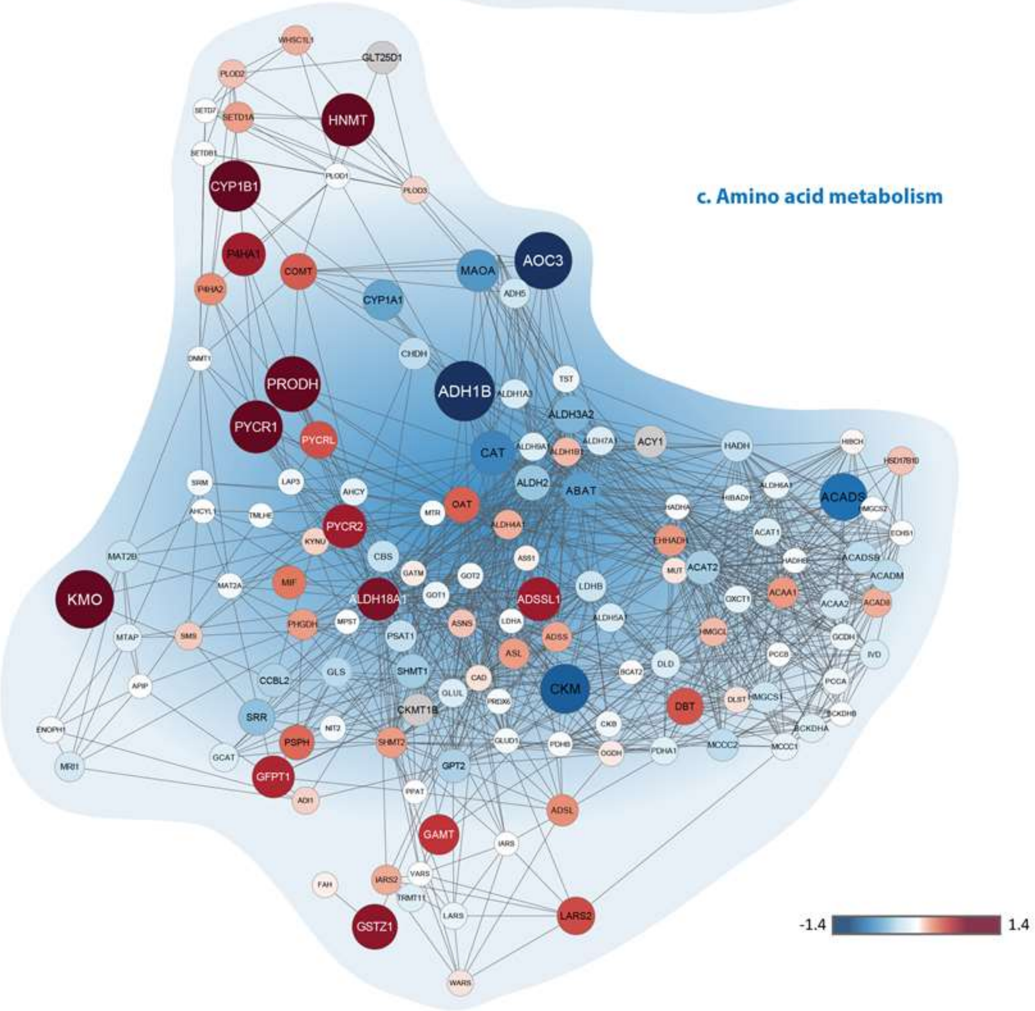

d. Translation

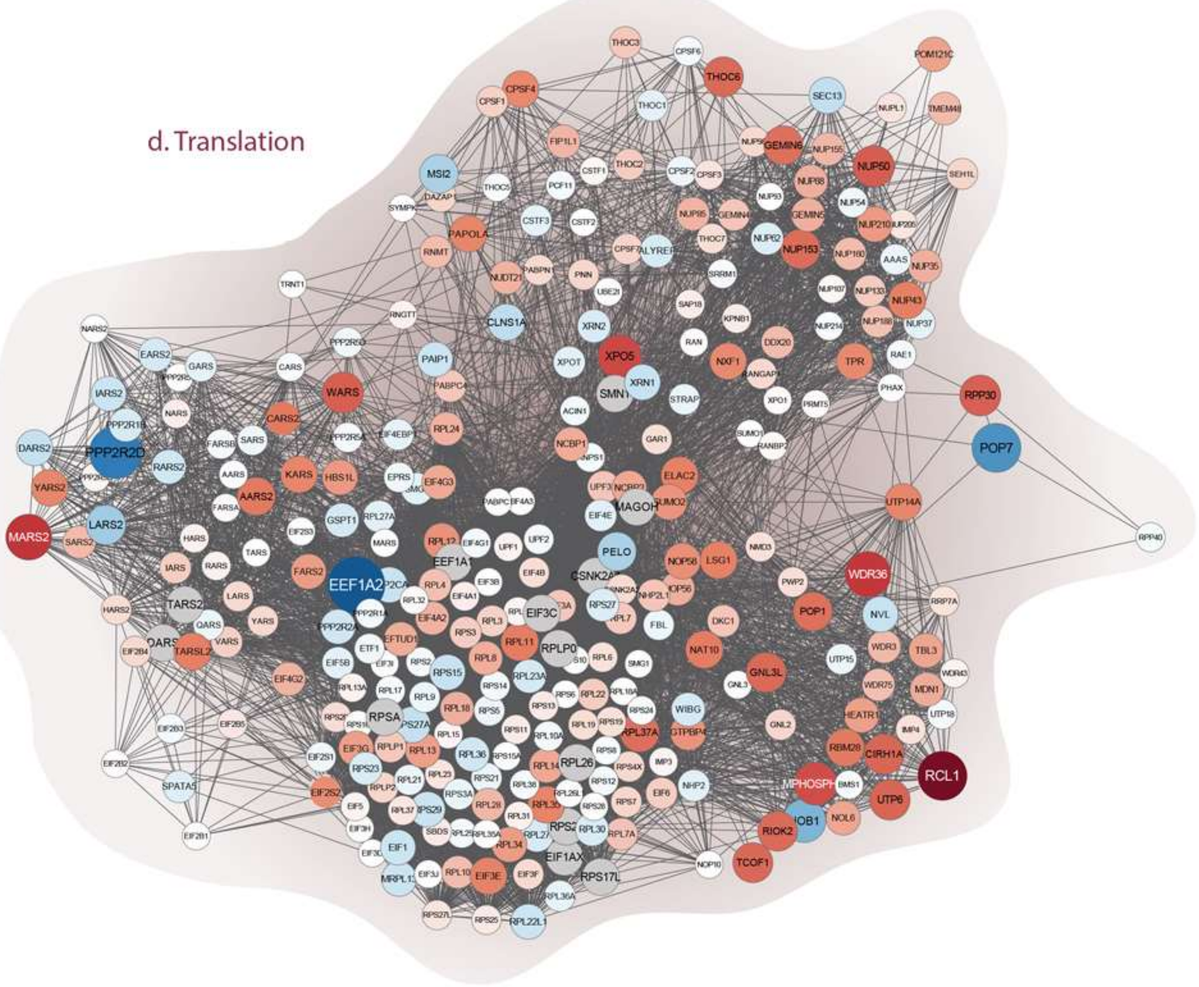

e. Replication

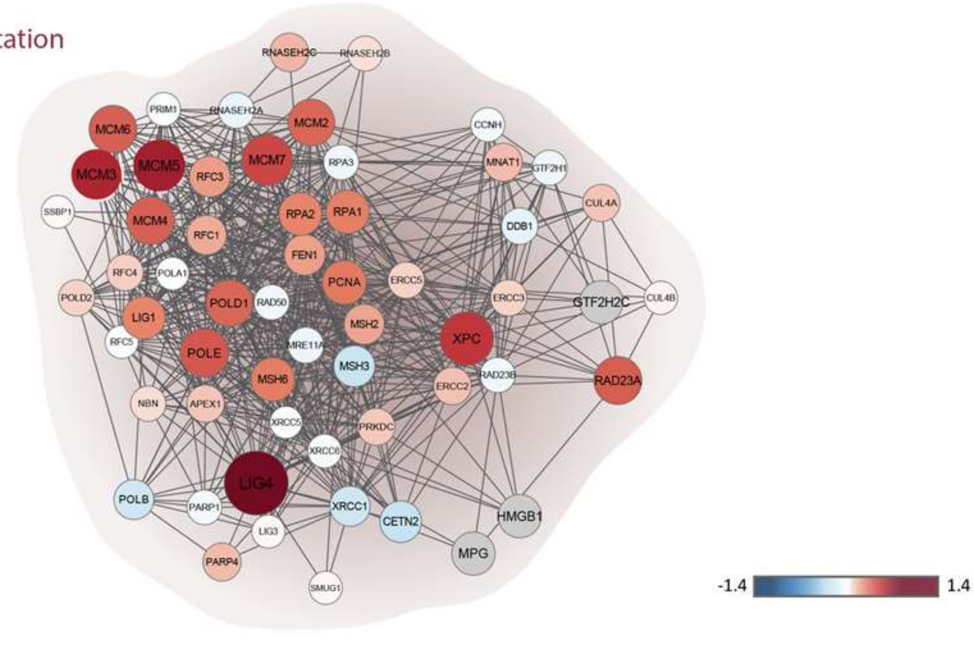

f. Cell growth and death

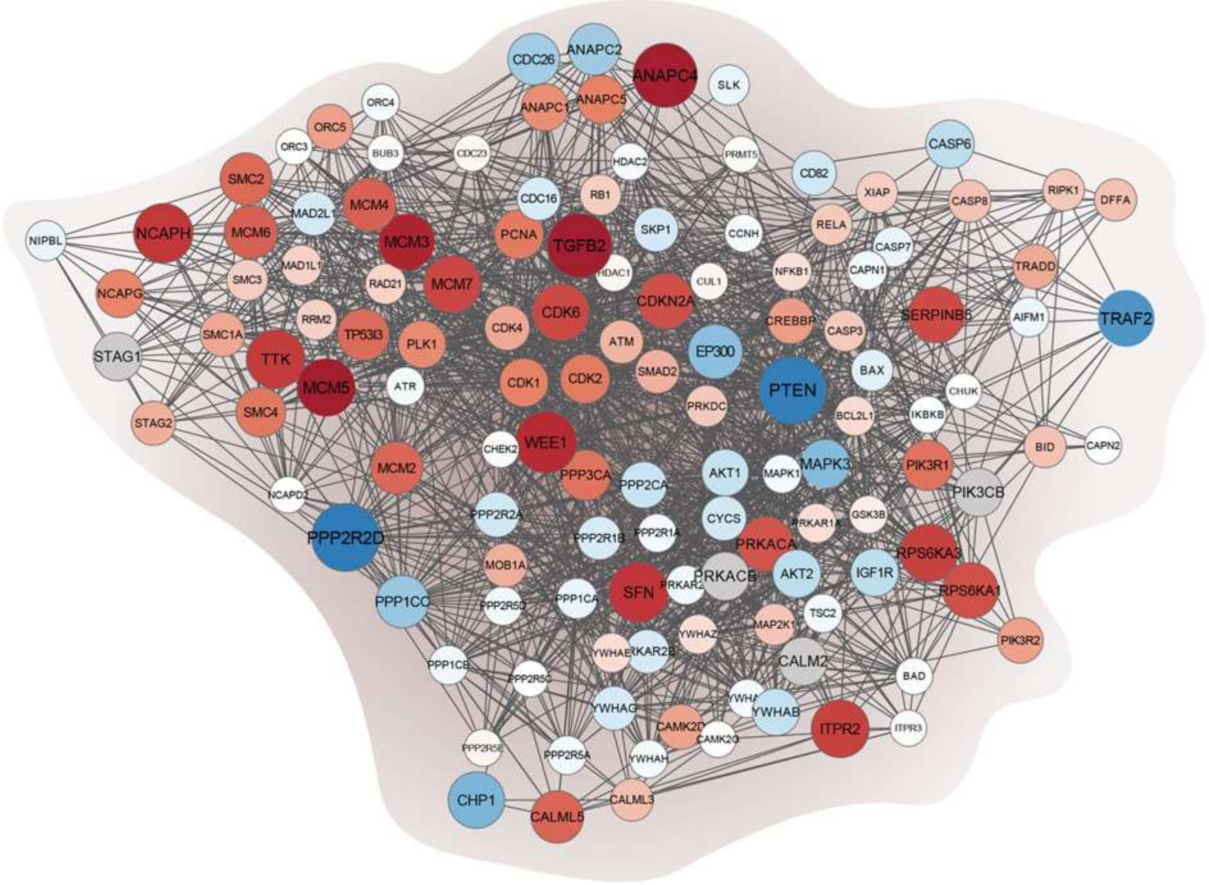

Supplementary Figure 4. Protein-protein interaction networks of enriched KEGG categories with differential subtype expression. The networks were constructed in String (www.string-db.org). The color of the nodes corresponds to the protein expression fold change between a particular subtype and the two other subtypes; red indicates higher expression and blue – lower expression in the selected subtype. The size of the nodes corresponds to the absolute protein expression fold change. **a**. Glycan biosynthesis and metabolism has higher average expression in the Her2 subtype relative to the ERPR and TN; Cellular community **(b)** and Amino acid metabolism **(c)** KEGG categories with lower average expression in the Her2 subtype; Translation **(d)**, Replication **(e)** and Cell growth and Death **(f)** KEGG categories with higher average expression in the TN subtype relative to ERPR and Her2.

Supplementary Figure 5

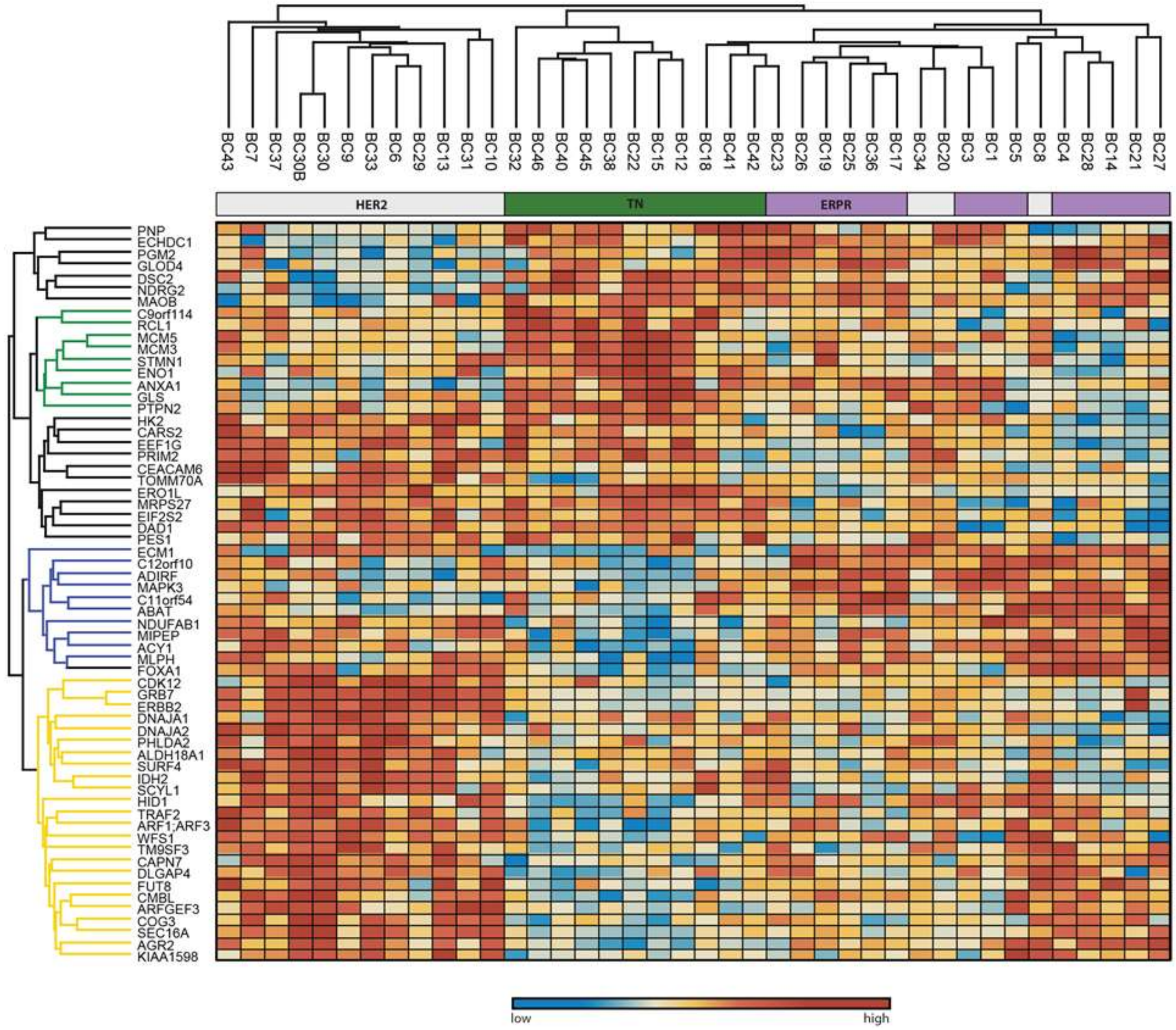

Supplementary Figure 5. Hierarchical clustering of differentially-expressed proteins. Differentially expressed proteins between the three cancer subtypes were identified with ANOVA analysis and permutation-based FDR of 5%. The proteins are colored according to their expression level. Subtype-specific clusters can be distinguished marked in (yellow: Her2, green: TN and blue: ERPR) on the row cluster tree. Cross-subtype clusters are shown in (black).

Supplementary Figure 6

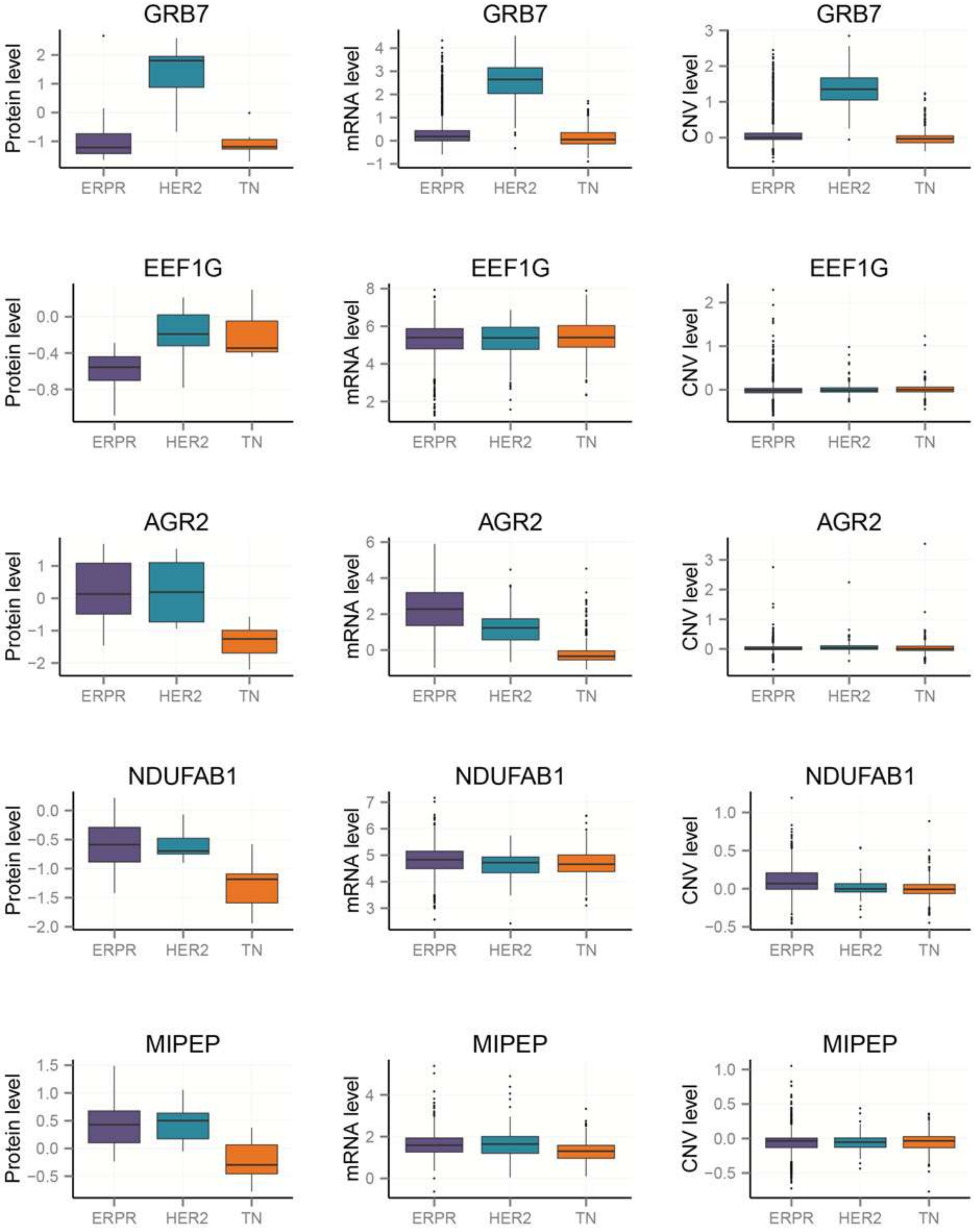

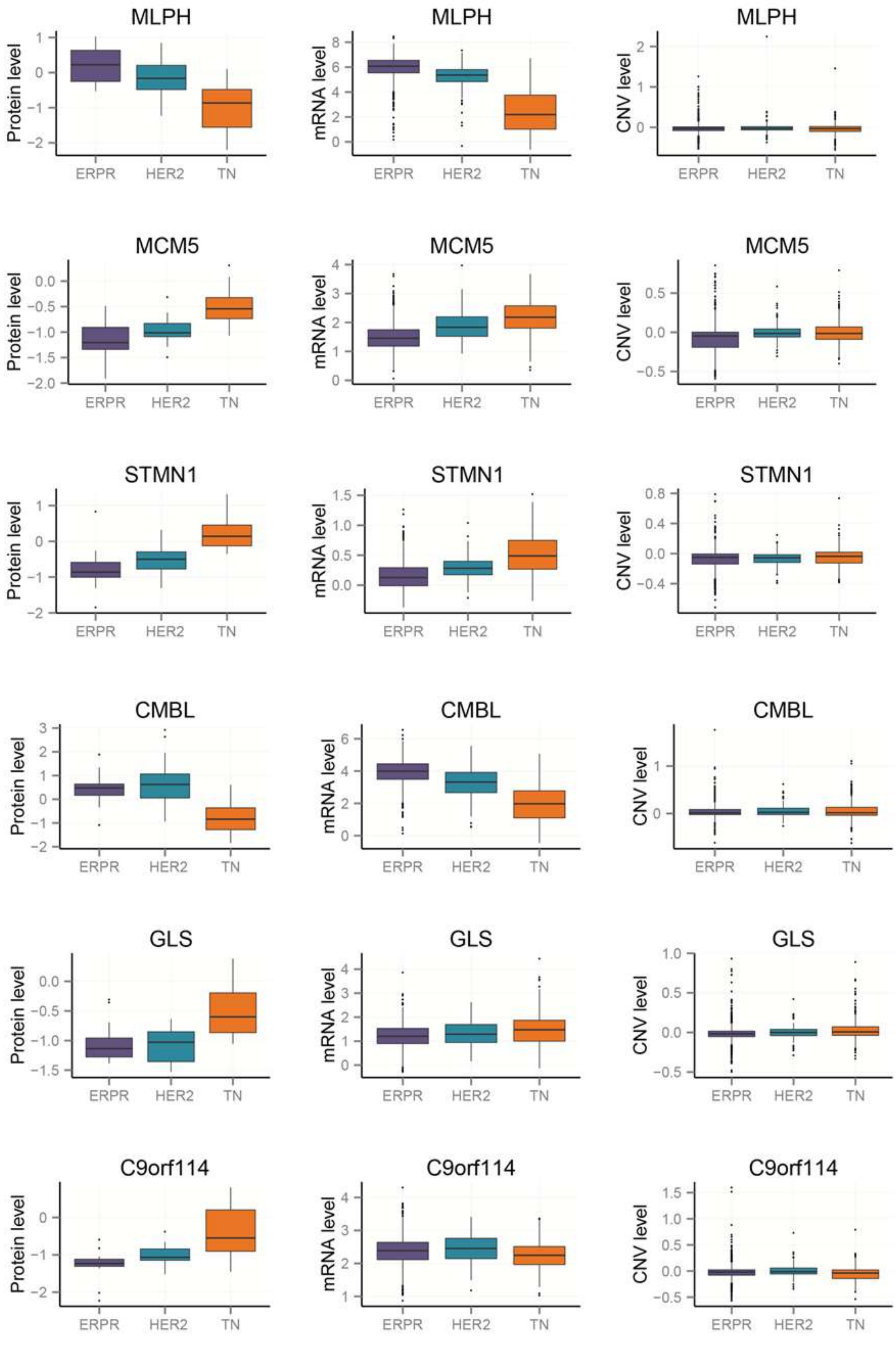
MLPH
Protein level
mRNA level
CNV level
MCM5
STMN1
CMBL
GLS
C9orf114
ERPR
HER2
TN

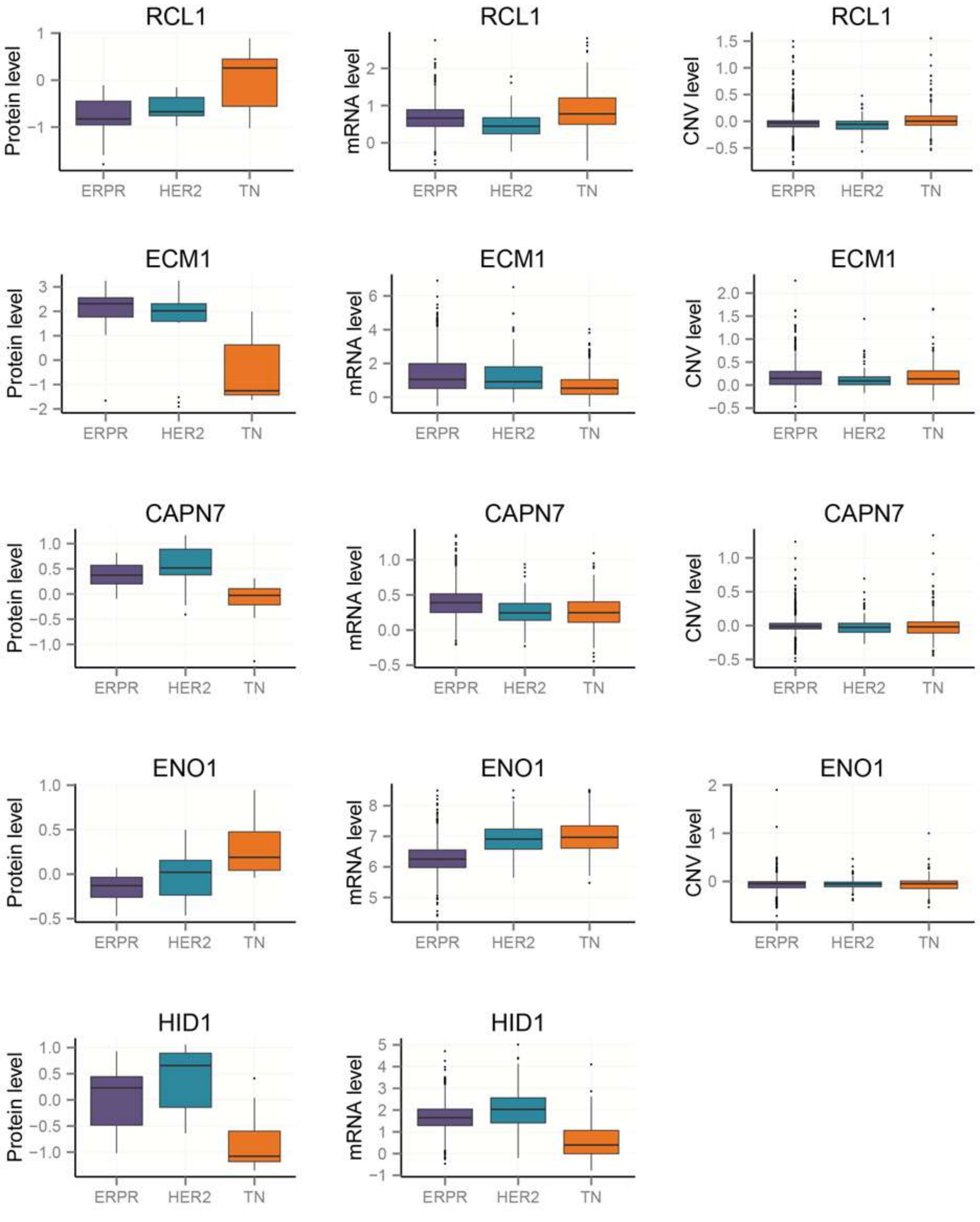

Supplementary Figure 6: Comparison of protein expression, mRNA expression and CNV distributions in each subtype for all signature proteins (excluding those shown in Figure 6)**.**

Supplementary Table 1

Sample #	Clinical classification	TNM stage	Grade	Type
1	ERPR	T1bN0M0	1	IDC
3	ERPR	T2N1M0	3	IDC
4	ERPR	T1cN0M0	2	IDC
5	ERPR	T1aN0M0	2	IDC
6	HER2	T2N1M0	3	IDC
7	HER2	T2N0M0	3	IDC
8	HER2	T1bN0M0	3	IDC
9	HER2	T2N0M0	2	IDC
10	HER2	T2N1M0	3	IDC
12	TN	T2N0M0	3	IDC
13	HER2	n.d	3	IDC
14	ERPR	n.d	1	IDC-Tu
15	TN	T2N0M0	3	IDC
17	ERPR	T2N1M0	3	IDC
18	TN	T2N0M0	3	IDC
19	ERPR	T2N1M0	3	IDC
20	HER2	T2N1M0	2	IDC
21	ERPR, Her2	T1cN1M0	3	IDC
22	TN	T2N0M0	3	IDC
23	ERPR	T1cN1M0	2	Lo
25	ERPR	T1cN1M0	2	IDC
26	ERPR	T2N1M0	2	Lo
27	ERPR	T2N0M0	3	IDC
28	ERPR	T1cN1M0	2	Lo
29	HER2	T2N1M0	2	IDC
30	HER2	T2N0M0	3	IDC
30B	HER2	T4N0M0	3	IDC
31	HER2	T2N2M0	3	IDC
32	TN	T2N1M0	3	IDC
33	HER2	T2N1M0	3	IDC
34	HER2	T2N1M0	3	IDC
36	ERPR, Her2	T2N0M0	3	IDC
37	HER2	T1cN0M0	3	IDC
38	TN	T1cN0M0	3	IDC
40	TN	T2N1M0	3	IDC
41	TN	T2N0M0	3	IDC
42	TN	T1cN0M0	3	IDC
43	HER2	T2N3M0	3	IDC
45	TN	T1cN0M0	3	IDC
46	TN	T2N0M0	3	IDC

Supplementary Table 1. Description of clinical samples. Information includes the tumor type, clinical subtype, tumor grade and stage for each tumor.
